# Iron (hydr)oxide formation in Andosols under extreme climate conditions

**DOI:** 10.1038/s41598-023-29727-1

**Published:** 2023-02-16

**Authors:** Björn Klaes, Sören Thiele-Bruhn, Gerhard Wörner, Carmen Höschen, Carsten W. Mueller, Philipp Marx, Helge Wolfgang Arz, Sonja Breuer, Rolf Kilian

**Affiliations:** 1 Geology Department, Trier University, Campus II (Geozentrum), Behringstraße 21, 54296 Trier, Germany; 2 Soil Science Department, Trier University, Campus II (Geozentrum), Behringstraße 21, 54296 Trier, Germany; 3 Division of Geochemistry and Isotope Geology, GZG, Georg-August-University Göttingen, Goldschmidtstraße 1, 37077 Göttingen, Germany; 4 Soil Science, Research Department Life Science Systems, TUM School of Life Sciences, Technical University of Munich, Emil-Ramann-Straße 2, 85354 Freising-Weihenstephan, Germany; 5 Department for Geosciences and Environmental Management, University of Copenhagen, Øster Voldgade 10, 1350 København K, Denmark; 6 Marine Geology Section, Leibniz Institute for Baltic Sea Research Warnemünde (IOW), Seestraße 15, 18119 Rostock, Germany; 7 Federal Institute for Geosciences and Natural Resources (BGR), Stilleweg 2, 30655 Hannover, Germany; 8 University of Magallanes, Avenida Bulnes 01855, Punta Arenas, Chile

**Keywords:** Biogeochemistry, Environmental sciences, Geochemistry, Mineralogy, Sedimentology, Volcanology

## Abstract

Redox-driven biogeochemical cycling of iron plays an integral role in the complex process network of ecosystems, such as carbon cycling, the fate of nutrients and greenhouse gas emissions. We investigate Fe-(hydr)oxide (trans)formation pathways from rhyolitic tephra in acidic topsoils of South Patagonian Andosols to evaluate the ecological relevance of terrestrial iron cycling for this sensitive fjord ecosystem. Using bulk geochemical analyses combined with micrometer-scale-measurements on individual soil aggregates and tephra pumice, we document biotic and abiotic pathways of Fe released from the glassy tephra matrix and titanomagnetite phenocrysts. During successive redox cycles that are controlled by frequent hydrological perturbations under hyper-humid climate, (trans)formations of ferrihydrite-organic matter coprecipitates, maghemite and hematite are closely linked to tephra weathering and organic matter turnover. These Fe-(hydr)oxides nucleate after glass dissolution and complexation with organic ligands, through maghemitization or dissolution-(re)crystallization processes from metastable precursors. Ultimately, hematite represents the most thermodynamically stable Fe-(hydr)oxide formed under these conditions and physically accumulates at redox interfaces, whereas the ferrihydrite coprecipitates represent a so far underappreciated terrestrial source of bio-available iron for fjord bioproductivity. The insights into Fe-(hydr)oxide (trans)formation in Andosols have implications for a better understanding of biogeochemical cycling of iron in this unique Patagonian fjord ecosystem.

## Introduction

Natural Fe-(hydr)oxide (trans)formation processes are crucial reactions in both terrestrial and aquatic/marine environments with significant implications for, e.g., a deeper understanding of biogeochemical cycles^1,2^, the reconstruction of paleoenvironmental conditions^3^, the genesis of banded iron formations^4^, and even extraterrestrial exploration^5^. In soils and sediments, such secondary Fe-phases occur predominantly as oxides, hydroxides and oxyhydroxides that (trans)form along abiotic or biotic pathways^6^. Amongst others, they comprise ferrihydrite (Fe_10_O_14_(OH)_2_), goethite (α-FeOOH), lepidocrocite (γ-FeOOH), hematite (α-Fe_2_O_3_), maghemite (γ-Fe_2_O_3_), and magnetite (Fe_3_O_4_), differing in thermodynamic stability and crystallinity^7^.

Fe-(hydr)oxides precipitate from solutions containing ferrous (Fe^2+^) and/or ferric (Fe^3+^) iron, dissolve or (re)precipitate from Fe-bearing minerals, or constitute pseudomorphs, (trans)formed from metastable precursor phases^7^. The growth mechanism during Fe-(hydr)oxide precipitation is principally defined by a nucleation-based aggregation pathway, in which the initial nano-scale compounds transform into thermodynamically stable crystallization products^8,9^. However, the nature and abundance of these Fe-(hydr)oxides depends on the bio-physicochemical and thermodynamic properties of the environment in which they are (trans)formed^6^. Consequently, the boundary conditions for the precipitation and growth of specific Fe-(hydr)oxides are defined by the dynamic interaction of various controlling parameters^7^. This interaction is complex and includes the interplay between the mineralogical/geochemical composition of parent materials or distinct precursor minerals with the aqueous phase, abundant organic matter (OM), iron-oxidizing/-reducing microorganisms and different temperatures under the prevalent redox-pH conditions^2,10,11^.

Iron (hydr)oxides play an important role in carbon sequestration and in biogeochemical cycles by the fixation or mobilization of iron and other essential bio-available elements for, e.g., the nutrient status of terrestrial ecosystems^2,12,13^ or the regulation of marine primary productivity^14^. Volcanic ash soils that evolved in humid environments can release high amounts of Fe- and OM-rich colloids^15,16^, while the element transport by such colloids sourced from peatlands represent key mechanisms for the nutrient supply in coastal regions and fjords of mid- and high-latitudes^17–19^. In particular peaty Andosols from the Magellanic moorlands are characterized by special element mobilization processes including the pronounced liberation of Fe-(hydr)oxides and OM under hyper-humid climate conditions^20^. Here, in the core zone of the southern westerly windbelt^21^ (SWW), variable and extraordinary high rainfall^22^ directly influences water-level fluctuations in sandy Andosol substrates^20^. In such dynamic biogeochemical-hydrological environments, the reactivity of organic and inorganic compounds are initially maintained by the abundance of redox-active metastable phases^23^ (RAMPs). The abundance of RAMPs also regulates ecosystem responses across scales, such as nutrient cycling and gas emissions^12,23^ (e.g., CO_2_, CH_4_ and N_2_O).

**Figure 1:**
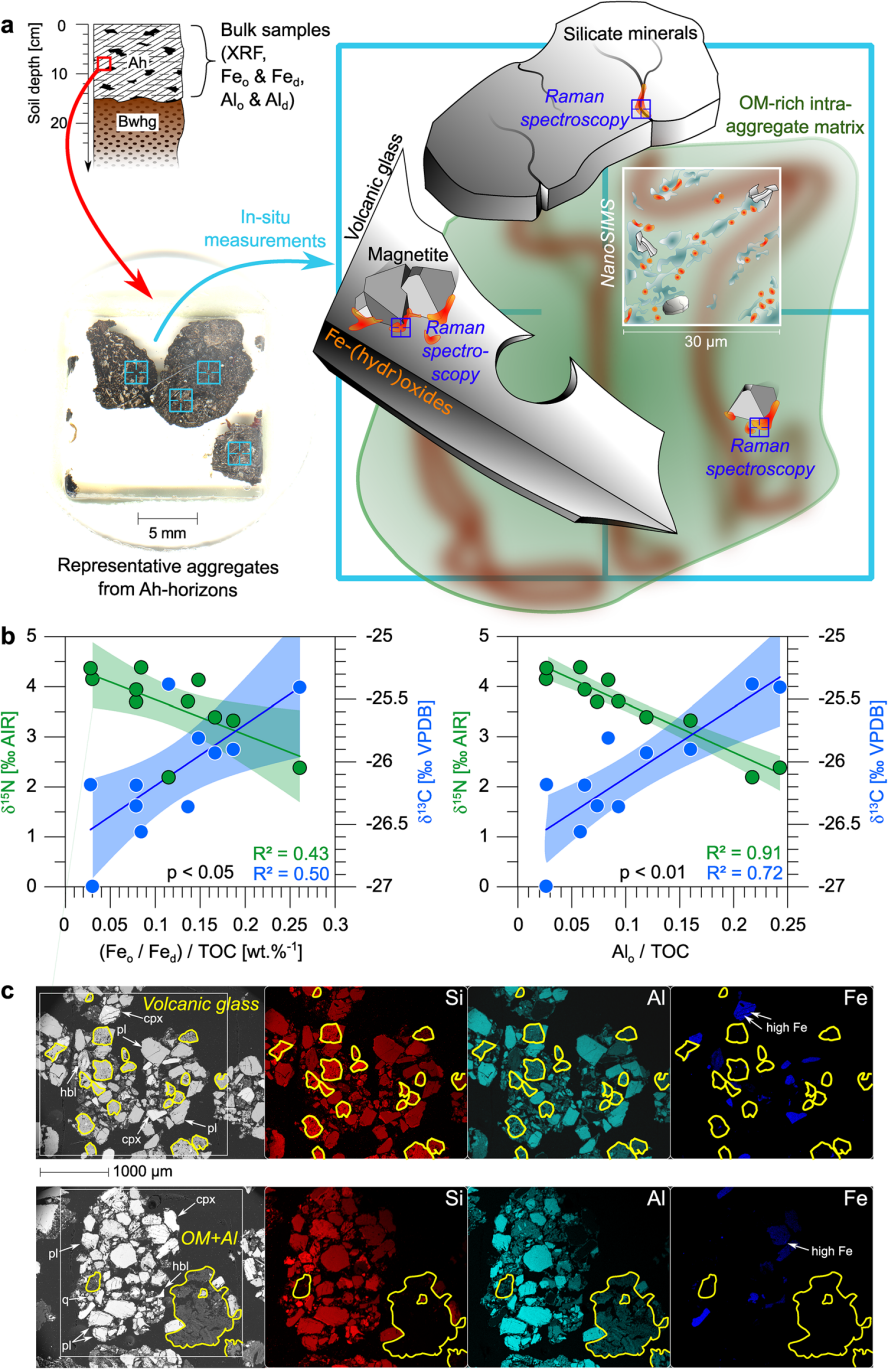
Introduction of the multiple-scale approach combined with the physico-geochemical boundary conditions for (hydr)oxide (trans)formation. (**a)** Schematic illustration of the study design encompassing bulk geochemical analyses and in-situ measurements on selected aggregates performed by Raman imaging spectroscopy and NanoSIMS. (**b)** (Fe_o_/Fe_d_)/TOC and Al_o_/TOC plotted versus δ^13^C and δ^15^N values from eleven bulk topsoil samples indicate the relationships between Fe-(hydr)oxide crystallinity/amorphous Al-(hydr)oxides and organic matter turnover. Subscripts refer to the treatments used for wet-chemical extraction (citrate bicarbonate dithionite—Fe_d_, ammonium oxalate—Fe_o_; Al_o_). Shaded fields represent 95% confidence intervals. (**c**) SEM micrographs showing the distribution of intra-aggregate components and SEM–EDS element mappings of Si, Al and Fe of representative topsoil aggregates. Residual volcanic glass and Al-humus complexes (OM + Al) are highlighted in yellow. Silicate minerals (*q* quartz, *pl* plagioclase, *cpx* clinopyroxene, *hbl* hornblende) and Fe-rich inclusions are marked.

However, hitherto little is known about the potentially important role of the terrestrial iron cycling in volcanic ash soils of the Magellanic moorlands and the land-to-fjord mass transfer of iron^20^. Therefore, we investigate Fe-(hydr)oxides formed at the redox interface in Ah-horizons from Andosols developed in this pristine South Patagonian peatland ecosystem that is affected by frequent and extreme hydrological disturbances. With this study we aim to identify mechanisms of Fe-(hydr)oxide (trans)formation from the weathering of rhyolitic tephra and evaluate their implication for the biogeochemical cycling in peaty Andosols to better constrain the land-to-fjord mass transfer of iron in this sensitive fjord region. Our approach includes bulk geochemical analyses using X-ray fluorescence (XRF) spectroscopy as well as wet-chemical pedogenic (hydr)oxide extractions. These bulk soil data were combined with micrometer-scale investigations of Fe-precipitates (Fig. 1a) in soil aggregates using scanning electron microscopy (SEM) with energy dispersive X-ray spectrometry (EDS), confocal Raman imaging spectroscopy and Nanoscale secondary ion mass spectrometry (NanoSIMS). We test the hypothesis that Fe-(hydr)oxide (trans)formation, the fixation of iron at redox interfaces as well as the provision of potentially bio-available Fe-compounds are regulated by oscillating redox conditions controlled by the most variable climate conditions. This should provide a better understanding of the biogeochemical cycling and mobilization of iron at hyper-humid active continental margins.

## Andosol characteristics and regional climate

In the small catchment at the Marcelo Arévalo site (52°41.7´S/73°23.3´W, ca. 650 m^2^ at 80 m a.sl.) situated in the extended Strait of Magellan fjord network, 45 cm thick Andosols developed in the reworked MB_2_ tephra deposit from Mt. Burney volcano^20^. Smaller but still significant amounts of volcanic ash have been deposited in the course of other, minor eruptive events during the Holocene^24–26^. The studied Dystric Hydric (Epileptic) Vitric Andosols were described in detail by Klaes et al.^20^ and their observations are briefly reported here: These soils have a sandy texture and are characterized by strong andolization since ~ 2.5 kyrs BP, involving the substantial chemical breakdown of volcanic glass and the simultaneous formation of Fe-/Al-(hydr)oxides. These processes are most pronounced in the organic-rich Ah-horizons, on which peat-forming plants grow (e.g., *Astelia*, *Cyperacea* and *Sphagnum* mosses). The Ah-horizons are acidic (pH 3.9–4.5) and have highly variable water contents (33–85 wt.%). They contain up to ~ 21 wt.% total organic carbon (TOC) and can release high amounts of dissolved organic carbon (DOC, up to 146 g kg^−1^ soil). Relative to unweathered MB_2_ tephra, these uppermost horizons are strongly enriched in Fe (up to 5.4 wt.% Fe_2_O_3_t) and Al (up to 22.4 wt.% Al_2_O_3_), comprising the Ti-bearing maghemite, hematite, and ferrihydrite, inferred from X-ray diffractomery (XRD). Titanomagnetite, which is typically abundant in pristine MB_2_ tephra, is absent. Secondary phases of Si and Al are amorphous and clay production is limited in these non-allophanic Andosols.

The hyper-humid climate at the site is largely controlled by the strength and position of the SWW^21,22^. Between 2010 and 2016, the nearby automatic weather station Arévalo recorded mean annual temperatures at 5.8 °C and annual rainfall of ~ 3800 mm year^−1^, whereby the seasonal rainfall is highly variable with highest precipitation rates during austral summer^27^. The local rainfall patterns typically include frequent extreme storm events with 20 mm day^−1^ precipitation or more^27^ (Fig. S1).

## Results

### Geochemistry and pedogenic oxide concentrations of bulk samples

The major element compositions and contents of Rb and Sr of five depth levels from a representative Ah-horizon are shown in Table 1. Compared to the other samples, the sample from 4 to 11 cm depth has lower contents of SiO_2_ (57.63 wt.%), K_2_O (1.03 wt.%) and Rb (22 mg kg^−1^), while TiO_2_ (1.39 wt.%), Al_2_O_3_ (16.86 wt.%), MgO (4.42 wt.%), CaO (5.54 wt.%), P_2_O_5_ (0.15 wt.%) and Fe_2_O_3_t (9.88 wt.%) are significantly higher. In general, the Sr concentrations increase continuously from 278 to 468 mg kg^−1^ towards the surface. With a value of 0.05, the sample from 4 to 11 cm depth shows the lowest Rb/Sr ratio.

**Table 1:** Major element compositions and Rb and Sr contents within an Ah-horizon from the MA catchment, obtained from XRF spectroscopy.

Depth (cm)	SiO_2_ (wt.%)	TiO_2_ (wt.%)	Al_2_O_3_ (wt.%)	Fe_2_O_3_t (wt.%)	MnO (wt.%)	MgO (wt.%)	CaO (wt.%)	Na_2_O (wt.%)	K_2_O (wt.%)	P_2_O_5_ (wt.%)	LOI (wt.%)	Total (wt.%)	Rb (mg kg^−1^)	Sr (mg kg^−1^)	Rb/Sr (mg kg^−1^)
0–4	70.46	0.86	13.92	4.38	0.08	1.96	3.51	3.36	1.41	0.07	26.43	100	31	476	0.07
4–11	57.63	1.39	16.86	9.88	0.11	4.42	5.54	2.99	1.03	0.15	19.45	100	22	468	0.05
11–15	68.54	0.91	14.55	5.75	0.07	2.53	2.62	3.57	1.38	0.07	14.25	100	36	359	0.10
15–19	68.76	0.85	14.72	5.81	0.10	2.68	2.43	2.76	1.81	0.08	14.48	100	58	254	0.23
19–22	67.04	1.01	15.42	6.01	0.10	2.91	2.68	2.86	1.86	0.10	13.54	100	62	278	0.23

Major elements are presented LOI-free and normalized to 100 wt.%. Low silica content and simultaneously reduced Rb/Sr ratios indicate the particular dissolution of volcanic glass^20^.

The concentrations of Fe and Al extracted by ammonium oxalate (subscript o, noncrystalline phases) and citrate bicarbonate dithionite (subscript d, noncrystalline plus crystalline phases) from eleven topsoil samples are listed in Table 2. The contents of extracted Fe are highly variable and range from 73 to 1267 mg kg^−1^ (Fe_o_) and from 90 to 2320 mg kg^−1^ (Fe_d_). The corresponding values of Fe_o_/Fe_d_ ratios lie between 0.48 and 0.87. The highest extractable element concentrations were obtained for Al_o_ (4106–7151 mg kg^−1^), while Al_d_ contents are considerably lower (0–1004 mg kg^−1^). Furthermore, samples showing very high Fe_o_ concentrations are also rich in TOC and have elevated δ^15^N values greater than 4‰.

**Table 2:** Pedogenic oxide concentrations of Fe and Al in eleven topsoil samples (0–5 cm depth).

	Fe_o_ (mg kg^−1^)	Fe_d_ (mg kg^−1^)	Fe_o_/Fe_d_	Al_o_ (mg kg^−1^)	Al_d_ (mg kg^−1^)	TOC* (wt.%)	TN* (wt.%)	δ^13^C* (‰ VPDB)	δ^15^N* (‰ AIR)
Topsoil1	1267	2320	0.55	4742	0	18.14	0.68	−27.00	4.15
Topsoil2	161	338	0.48	4437	498	6.04	0.27	−26.35	3.70
Topsoil3	188	384	0.49	4566	1004	1.88	0.09	−25.41	2.38
Topsoil4	231	314	0.74	6299	677	3.94	0.16	−25.90	3.32
Topsoil5	102	270	0.38	7151	116	3.29	0.14	−25.38	2.19
Topsoil6	133	171	0.78	5323	0	5.71	0.26	−26.36	3.71
Topsoil7	634	725	0.87	5994	0	10.37	0.46	−26.56	4.38
Topsoil8	551	707	0.78	4406	535	5.26	0.24	−25.81	4.14
Topsoil9	73	90	0.81	5780	71	4.85	0.23	−25.93	3.39
Topsoil10	153	292	0.52	4106	0	6.64	0.33	−26.19	3.94
Topsoil11	416	704	0.59	5588	0	21.06	0.94	−26.18	4.37

Organic geochemical and isotope data are obtained from Klaes et al.^20^ (marked with *). Subscripts refer to the treatments used for wet-chemical extraction (citrate bicarbonate dithionite—Fe_d_, Al_d_; ammonium oxalate—Fe_o_, Al_o_).

The scatter plots (Fig. 1b) relating the (Fe_o_/Fe_d_)/TOC and Al_o_/TOC ratios and stable isotope data (δ^13^C, δ^15^N) are used to assess the possible complexation of OM with Fe and Al as a function of Fe-(hydr)oxide crystallinity, the content of amorphous Al-(hydr)oxides, and the turnover degree of associated OM. In both cases, the ratios decrease with lower δ^13^C values and higher δ^15^N, indicating the presence of strongly decomposed OM^20^. The correlation between (Fe_o_/Fe_d_)/TOC and stable isotope data is weak to moderate (R^2^ = 0.43 for δ^15^N; R^2^ = 0.50 for δ^13^C), while correlation of Al_o_/TOC values with δ^13^C is strong (R^2^ = 0.72) and with δ^15^N it is very strong (R^2^ = 0.91). However, significant correlations with δ^13^C and δ^15^N values are not observed for Fe_o_/TOC or Fe_d_/TOC (Fig. S2).

### Composition of soil aggregates and tephra

Back-scattered electron images and element mappings by EDS display the internal structure of six selected soil aggregates, their mineralogical composition, and the intra-aggregate distribution of Si, Al and Fe (examples in Fig. 1c). The silicate components are embedded in a matrix of OM and mostly comprised of phenocrysts (plagioclase, hornblende and clinopyroxene) from MB_2_ tephra. Other silicates (quartz, mica) are sourced from the granitic/gneissic basement rocks. Rare volcanic glass from MB_2_ tephra occurs scattered throughout the aggregates as residual small fragments (< 300 μm) after partial dissolution. Element mapping reveals that areas with high Fe-content appear either as isolated, micrometer-scale spots in the aggregates or are concentrated as inclusions in silicate components. Aluminum extensively occurs throughout the OM-rich matrix and is enriched at the margins of individual silicate grains. The relative proportions calculated from XRD diffractograms show that pristine MB_2_ tephra is composed of 70% volcanic glass, 18% plagioclase, 6.5% clinopyroxene, 3.5% hornblende and 2% titanomagnetite. The EDS measurements on 65 areas of interest (AOIs) of pumice particles from the MB_2_ layer in these soils demonstrate that titanomagnetite phenocrysts contain ~ 50.2 wt.% Fe on average and up to 27.4 wt.% Ti (Figs. S3 and S4) and are low in Si (> 4.7 wt.%), Al (> 1.7 wt.%) and Mg (> 1.5 wt.%). The MB_2_ glass surrounding these oxide grains contains < 1.8 wt.% Fe and is low in Ti (< 0.3 wt.%).

### Identification of secondary Fe-phases by confocal Raman imaging spectroscopy

**Figure 2:**
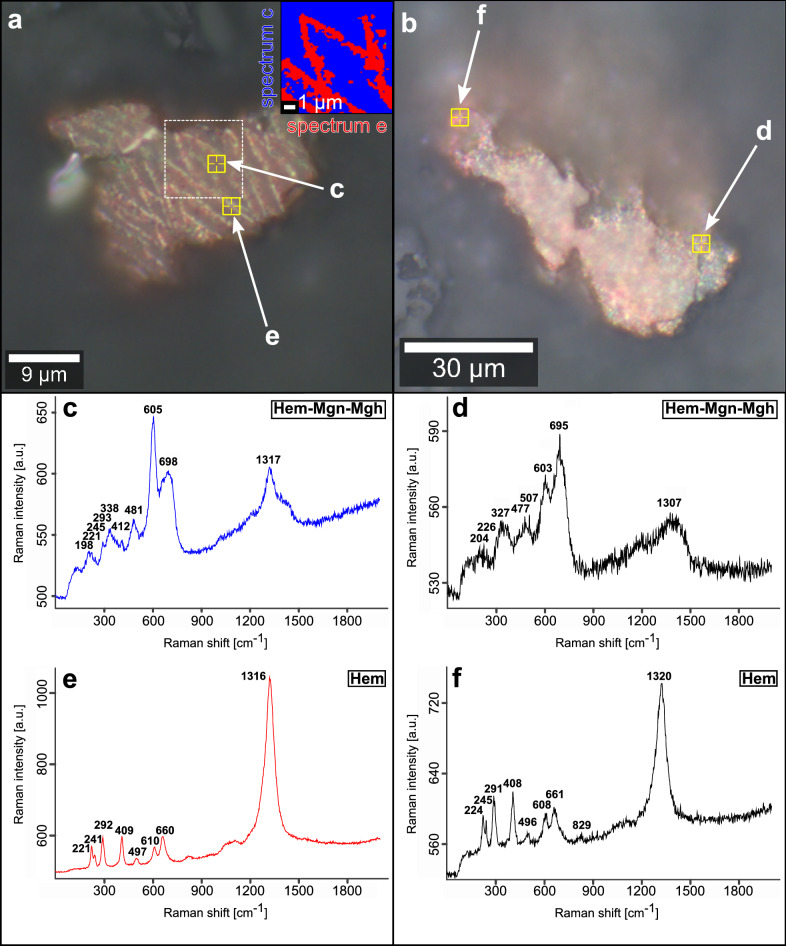
Raman spectra obtained from former MB_2_ titanomagnetite phenocrysts embedded in soil aggregates. **(a,b)** Reflected light microphotographs of individual grains with diameters of ~ 40 to 60 μm. The inset in (**a)** shows the false color image of the dominant Raman spectra (representing spectra **c,e**) determined upon area scan of the region marked with the white square. (**c,d**) Mixed spectra comprising spectral information of magnetite, maghemite and hematite. (**e,f**) Raman spectra of hematite. Bold numbers represent wavenumbers of diagnostic Raman bands. Yellow markings indicate the localizations of the respective measurements.

**Figure 3:**
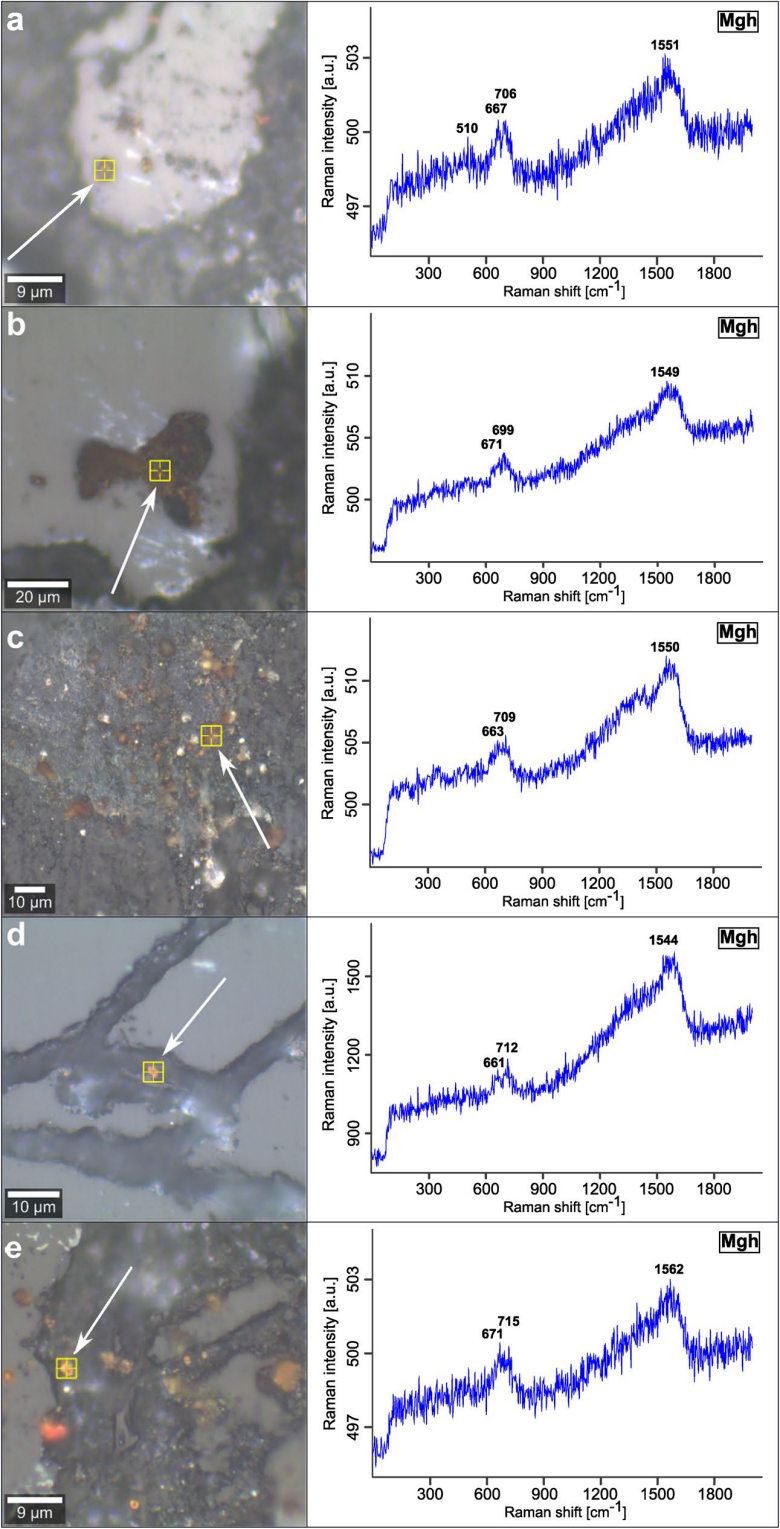
Raman spectra of maghemite deduced from micrometer-scale crystals embedded in soil aggregates. **(a–e**) Reflected light microphotographs in combination with Raman spectra of maghemite. Bold numbers represent the wavenumbers of diagnostic Raman bands. Yellow markings indicate the localizations of the respective measurements. The crystals shown are either situated in voids of MB_2_ glass (**a,b**), or occur dispersed within the organic-rich matrix of the aggregates (**c–e**).

**Figure 4:**
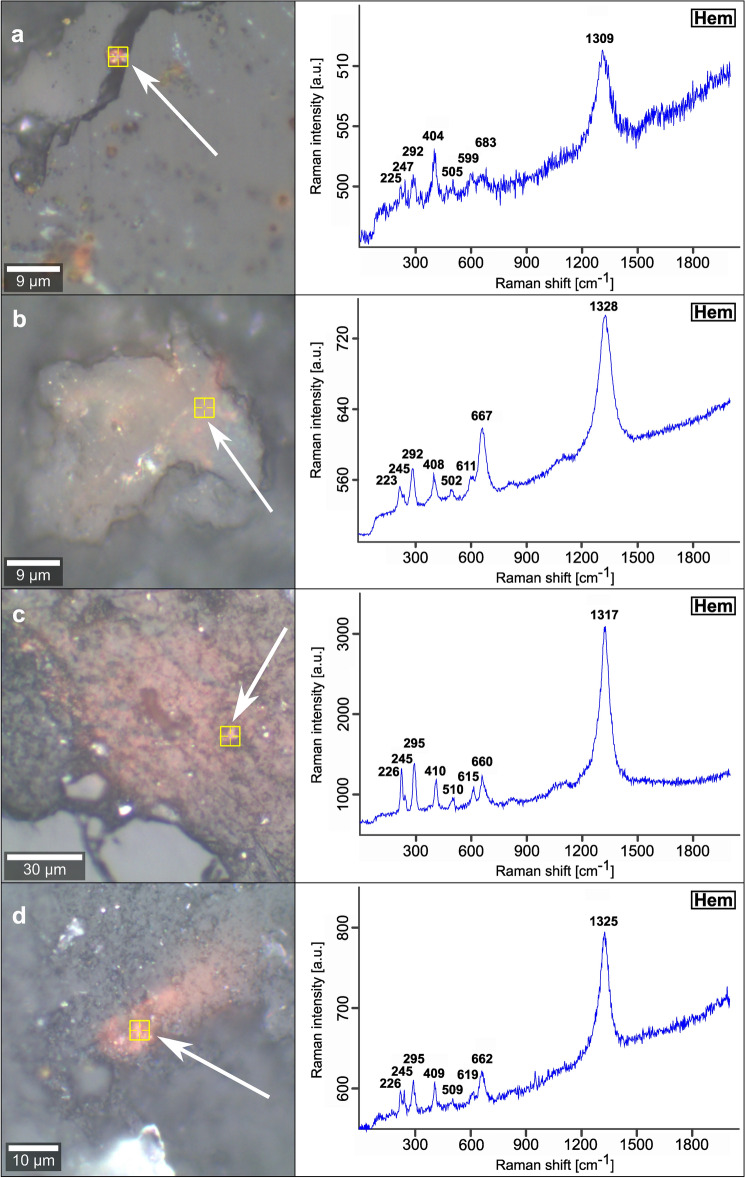
Raman spectra of hematite deduced from micrometer-scale crystals embedded in soil aggregates. **(a–d)** Reflected light microphotographs in combination with Raman spectra of hematite. Bold numbers represent the wavenumbers of diagnostic Raman bands. Yellow markings indicate the localizations of the respective measurements. Hematite precipitates have been either observed in small voids of silicate components (MB_2_ glass, (**a**) or form more extended Fe-(hydr)oxide crusts within the aggregates (**b–d**).

The Fe-(hydr)oxides on more than 250 AOIs in 50 soil aggregates and five altered sections of weathered granite are identified by Raman spectroscopy measurements (Figs. 2, 3 and 4, S5 and S6). The Raman spectra were evaluated according to published wavenumbers of diagnostic bands for Fe-(hydr)oxides (Table S1). Considering differences in the crystallinity of natural Fe-(hydr)oxides, the potential analytical bias of the equipment used, and limited spectral resolution, we accepted a discrepancy of ± 10 cm^−1^ between our data and the wavenumbers of diagnostic reference Raman bands^28,29^ (Table S1).

Measurements on grains of ~ 40 to 60 μm size (Fig. 2a,b) often gave mixed Raman spectra with overlapping signals of magnetite, maghemite and hematite (Fig. 2c,d). Some spectra identify pure hematite (Fig. 2e,f). For a selected micro-region on such a crystal, the spatial distribution of different Raman spectra is illustrated by a false color image derived from a cluster analysis of the Raman signals (Fig. 2a, insert). Here, the regions with a mixed spectrum (blue cluster) represents the matrix composition of this grain, whereas pure hematite (red cluster) is concentrated within this mixed domain in the form of linear and crossing structures. Maghemite crystals in soil aggregates are ~ 3 μm in diameter and were detected either in porous, residual MB_2_ glass (Fig. 3a,b) or are dispersed in the OM-rich matrix (Fig. 3c–e). Hematite precipitates occur as ~ 3 to 5 μm-sized crystals localized in small voids of silicate components, e.g., volcanic glass (Fig. 4a), or form more extensive Fe-(hydr)oxide crusts in the soil aggregates (Fig. 4c,d). In such crusts, hematite represents the sole Fe-(hydr)oxide identified by Raman spectroscopy. Micrometer-sized goethite crystals have not been found in soil aggregates. The occurrence of goethite is restricted to cracks within alteration rims of weathered basement rocks. There, it precipitates next to rare hematite (Fig. S5). However, we observed that many of our Raman spectra show broad bands at ~ 510 and especially at ~ 710 cm^−1^ (e.g., in Figs. 3 and 4). These bands typically serve as diagnostic criteria for the occurrence of ferrihydrite^30,31^. In addition to Fe-(hydr)oxides, few siderite crystals (Fe^2+^CO_3_; ~ 10 μm in diameter) were discovered in the OM-rich matrix close to residual MB_2_ glass (Fig. S6).

### NanoSIMS measurements

**Figure 5:**
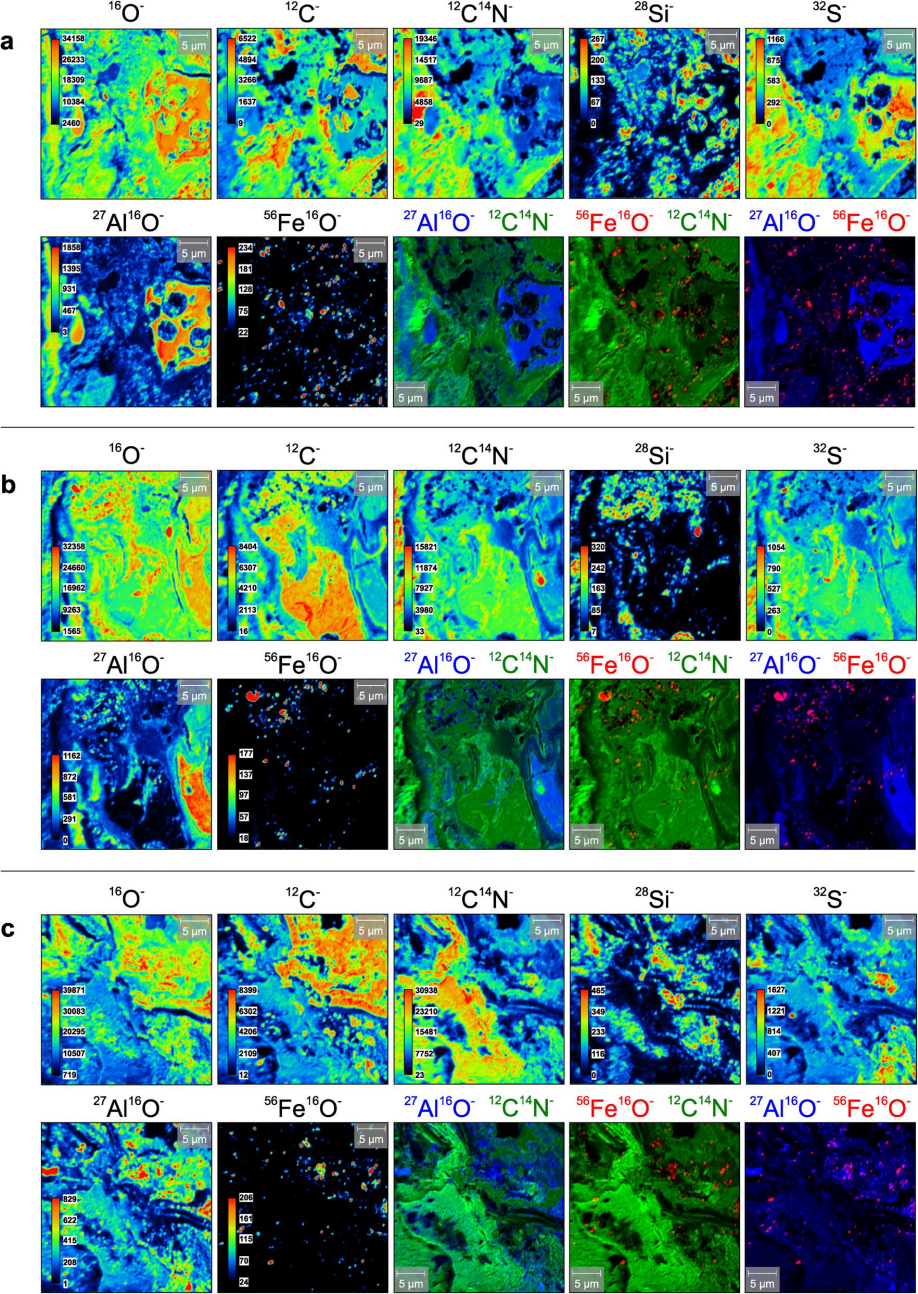
NanoSIMS secondary ion mappings of nano- to micrometer-scale intra-aggregate components. **(a–c**) Spatial distribution of ^16^O^−^, ^12^C^−^, ^12^C^14^N^−^, ^28^Si^−^, ^32^S^−^, ^27^Al^16^O^−^, and ^56^Fe^16^O^−^ secondary ions measured on 30 × 30 μm surfaces within the organic-rich matrix of the soil aggregates. In addition, composite images calculated from ^12^C^14^N^−^, ^27^Al^16^O^−^ and ^56^Fe^16^O^−^ data are shown.

We applied NanoSIMS to collect nanometer-scale information on the spatial distribution of organic and inorganic compounds within the soil aggregates (Figs. 5 and S7). In total 27 measurements on 30 × 30 μm AOIs were recorded and analyzed, which all showed similar patterns with respect to the spatial distribution of the secondary ion counts of O, C, N, S, Si, Al and Fe. Strongly correlating signals of ^12^C^−^ and ^12^C^14^N^−^ cover most of these regions and represent the intra-aggregate matrix dominated by peat plant-sourced OM. This intra-aggregate matrix contains patches of elevated ^28^Si^−^ and ^16^O^−^ counts, which are framed by irregularly shaped rims enriched with ^27^Al^16^O^−^. The ^28^Si^−^ enriched areas themselves show a low incidence of ^27^Al^16^O^−^. With increasing distance to these Si–Al–O-rich structures, ^27^Al^16^O^−^ gradually decreases and overlaps with secondary ion counts of ^12^C^−^, ^12^C^14^N^−^ and ^32^S^−^.

However, in distinct areas, ^27^Al^16^O^−^ and ^32^S^−^ are highly correlated. Contrasting with the widely distributed occurrence of ^27^Al^16^O^−^, distinct spots with localized high ^56^Fe^16^O^−^ secondary ion counts are dispersed in the matrix and form clearly defined, smaller clusters. These spots of similar shape are up to 3 μm in size and concentrate in areas that are also rich in ^28^Si^−^ and ^16^O^−^. Composite images calculated from ^12^C^14^N^−^, ^27^Al^16^O^−^ and ^56^Fe^16^O^−^ (Figs. 5 and S7) show that the spatial distribution of high ^56^Fe^16^O^−^ signals is neither correlated with elevated secondary ion counts of ^12^C^14^N^−^ nor with ^27^Al^16^O^−^. In fact, the composite images highlight the uniform distribution of ^27^Al^16^O^−^ and ^12^C^14^N^−^ signals that strongly contrast with the spatial pattern of Fe-rich spots.

## Discussion

The deposition of volcanic material into terrestrial and marine ecosystems represents a crucial source for essential elements for the functioning of a multitude of biogeochemical cycles^32^. This is due to the physical nature and composition of tephra pumice: pyroclastic particles are generally fine-grained, predominantly vitreous and therefore have a low resistance to chemical weathering^15^. Consequently, given the composition, size and high porosity of tephra, volcanic glass is the major source for the formation of noncrystalline secondary silicates and (hydr)oxides in Andosols^33^. Furthermore, such formation of secondary minerals is strongly aided by the high acidity in these soils and the interaction with accumulated OM^34,35^. The rapid chemical breakdown of glassy pumice primarily results in the release of Si, Al and Fe^16,36^. Accordingly, the relative depletion in SiO_2_, K_2_O, low Rb/Sr and simultaneously elevated Al_2_O_3_ and Fe_2_O_3_t contents are linked to the development of a distinct redox interface within the Ah-horizons (at 4 to 11 cm depth in the example given in Table 1). At this interface, the dissolution of MB_2_ glass and the subsequent formation of secondary phases is most pronounced^20^. Redox dynamics of Fe-(hydr)oxides are typically accompanied by similar dynamics of Mn-(hydr)oxides that are even more prone to redox changes^6,11^. However, Mn contents in primary and secondary minerals in basement rocks^20^, soils^20^ (Table 1, Figs. S3 and S4) and cave stalagmites^26^ from this area were low and rarely exceeded detection limits. Also based on previous findings, we suggest that the evolution of such redox interfaces in South Patagonian Andosols is caused by frequent water-table fluctuations^20,23^ modulated by the strong and SWW-driven variations in rainfall intensity^27^.

In such dynamic redox systems, organic acids and labile organic compounds originating from OM turnover are ubiquitous and stimulate Al-(hydr)oxide precipitation as the most important proton donors^35^. At pH values < 5, the Al^3+^ released by alteration of glass (Fig. 1c) in the uppermost soil columns preferentially forms noncrystalline colloidal phases as indicated by elevated Al_o_ concentrations relative to Al_d_^36^ (Table 2), e.g., Al_x_(OH)^3x-y^-humus complexes^33^. Here, the proportion of decomposed OM, which is also controlled by water-table fluctuations^37,38^, plays an important role due to its various functional groups (e.g., hydroxylic, carboxylic and phenolic acids^11^). Considering the relationship between changing Al_o_/TOC ratios with lower δ^13^C and higher δ^15^N values (Fig. 1b), we assume that Al-humus complexation is a function of (i) Al^3+^-supersaturation and (ii) the presence of a large pool of labile OM in soil solutions^20^. In addition, our NanoSIMS measurements (Figs. 5 and S7) demonstrate the occurrence of residual MB_2_ glass, represented by isolated areas high in ^28^Si^−^ and ^16^O^−^ secondary ion counts that are framed by irregularly shaped ^27^Al^16^O^−^ rims^26^. With a greater distance to the glass, ^27^Al^16^O^−^ merges gradually with ^12^C^−^ and ^12^C^14^N^−^, while spots with elevated ^28^Si^−^ and ^16^O^−^ counts remain isolated in the OM-rich matrix. We interpret this observation as a direct formation of Al-humus complexes from glass weathering, suppressing the coprecipitation of Al^3+^ with silica to form crystalline aluminosilicates (absent in these soils^20^). This then results in the precipitation of opaline silica/amorphous silica gels from H_4_SiO_4_^34^. Al-humus complexes can form vast structures in soil aggregates (Fig. 1c) and are primarily responsible for PO_4_^3−^ sorption processes in Andosols^33^, explaining the rise of P_2_O_5_ with Al_2_O_3_ at the redox interface (Table 1). We further suggest that the uniform distribution of ^12^C^−^, ^12^C^14^N^−^ and ^27^Al^16^O^−^ with ^32^S^−^ in the NanoSIMS mappings (Figs. 5 and S7) indicates that Al-humus complexes also represent the major colloidal constituent for the sorption of S-bearing species in these Ah-horizons.

Even though pristine MB_2_ glass contains 13.3 wt.% Al_2_O_3_ on average but only 1.4 wt.% Fe_2_O_3_t^26,39–41^, both elements show a correlated enrichment relative to bulk MB_2_ tephra in the uppermost horizons^20^. Amongst the previously identified Fe-(hydr)oxides, ferrihydrite is the most likely candidate to form from Fe^2+^ released after reductive dissolution^35^ of MB_2_ glass in the Ah-horizons. This metastable, short-range ordered Fe-(hydr)oxide occurs as nanoparticle, is known to adsorb high amounts of OM and amorphous silica in non-allophanic Andosols^15,35^, and is related to elevated Fe_o_ concentrations^16,36^. Unlike highly abundant Al-humus complexes (Al_o_/TOC in Fig. 1b), there is no evidence for a correlation of Fe_o_/TOC with δ^13^C or δ^15^N values (Fig. S2). Yet (Fe_o_/Fe_d_)/TOC ratios, reflecting the proportion of ferrihydrite in the total amount of Fe-(hydr)oxides relative to OM content, moderately correlate with δ^13^C or δ^15^N (Fig. 1b). We address this relationship to a decrease in iron associated with labile OM compounds related to changes in redox state (Fe^2+/3+^) and increasing Fe-(hydr)oxide crystallinity^10,42,43^, sharply contrasting with the behavior of aluminum. The spatial distribution of (sub)micrometer-scale Fe-(hydr)oxides within the soil aggregates can be attributed to this increase in crystallinity. NanoSIMS secondary ion mappings and composite images (Figs. 5 and S7) reveal that many dispersed ^56^Fe^16^O^−^ spots are associated with patchy enrichments in ^28^Si^−^ and ^16^O^−^ (residual glass or amorphous silica). The larger these spots enriched with ^56^Fe^16^O^−^ become, the stronger they are decoupled from siliceous matter and from areas with elevated ^12^C^−^, ^12^C^14^N^−^ and ^27^Al^16^O^−^ secondary counts (Figs. 5 and S7). We interpret this change in spatial correlation of ^12^C^−^ and ^12^C^14^N^−^ patterns with the size of ^56^Fe^16^O^–^ rich spots as the result from a shift from mostly microbially-mediated Fe-(hydr)oxide nucleation from Fe^2+/3+^-OM-associations^12,44,45^ towards a more abiotic, oxidation-promoted growth process.

Recent studies highlight that redox (trans)formations of iron in soils are controlled by a complex cascade of abiotic and biotic processes^2,6,11^. The reduction and oxidation of iron by microorganisms should be inseparably linked to the amount of organic ligands (OL) and DOC released from intense OM turnover^12,38,43^ as well as to the cycling of specific nutrients (especially N, P and S^10,46^). This suggests an efficient interaction of the iron redox cycling with the simultaneously formed Al-humus complexes and the dominant oxyanions adsorbed, e.g., NO_x_^−^, PO_4_^3−^, SO_4_^2−^. Furthermore, an iron redox cycling of this type requires a highly dynamic reciprocal action between dissolved species, microbial biomass, OL and RAMPs, resulting in a mechanism with a frequent exchange of electrons, which was previously called a biogeobattery^23^. Drastic changes in the redox conditions (i.e., different oxygen penetration depths) due to water-level fluctuations should strongly reinforce this biogeochemical cycling^13,37^.

Therefore, we suggest a crystallization pathway for ferrihydrite as nanoparticle from OM-rich precursor phases (noncrystalline RAMPs, Fe^2+/3+^ complexed with OL^47^) after Fe-(hydr)oxide nucleation induced by the rapid oxidation of OM-associated Fe^2+^ together with dissolved Fe^2+^ in soil solutions^8,48^ (Eq. 1). We expect that ongoing crystal growth from such Fe^2+/3+^-OM coprecipitates to ferrihydrite (+ OM) is determined by microbially-mediated dissolution-(re)crystallization processes^8,48^. This is consistent with other studies, indicating that the stability of iron complexed with OL is typically reduced in acidic Andosols^15^.

1 $${Fe}_{ dissolved}^{2+}\stackrel{+OL +microbes }{\Longrightarrow }{ }_{noncrystalline}RAMPs\stackrel{+{Fe}_{ dissolved}^{2+}+{H}_{2}O +{O}_{2} +microbes }{\Longrightarrow }{Fe}_{10}^{3+}{O}_{14}{(OH)}_{2}$$

2 $${Fe}_{10}^{3+}{O}_{14}{(OH)}_{2}\stackrel{+{Fe}_{ dissolved}^{2+}-{H}_{2}O +{O}_{2} +microbes}{\Longrightarrow }{Fe}_{2}^{3+}{O}_{3}$$

Hence, the so-formed ferrihydrite experiences dehydration under constant supply of dissolved Fe^2+^ which entails the (trans)formation to better crystalline and thermodynamically stable Fe-(hydr)oxides after further successive redox cycles^49,50^. Inferred from XRD data^20^ and our Raman spectroscopy measurements (Figs. 2 and 4), we consider hematite representing the most stable Fe-(hydr)oxide produced by such (trans)formation pathways in this acidic environment (Eq. 2). Hematite in particular—and no other Fe-(hydr)oxide—forms comparatively large-scale crusts within the soil aggregates (Fig. 4b–d), pointing towards a distinct and persistent accumulation at the redox interface due to its high thermodynamic stability^50^. By contrast, goethite crystallization is inhibited by the high saturation of DOC, dissolved silica and Al^3+^ in soil solutions of non-allophanic Andosols^33–35^. Therefore, goethite precipitates are restricted to microenvironments in weathered basement rocks (Fig. S6), where the influence of solutes from andolization is less pronounced. The particular setting of rapid glass dissolution and intense OM turnover is typically characterized by a considerable release of HCO_3_^−^^2,34,51^, which can significantly accelerate hematite (trans)formation from ferrihydrite^49^. Abundant aqueous Fe^2+^ along with HCO_3_^−^ is documented by the presence of siderite (Fig. S6), indicating significant shifts in pH values, variations in dissolved CO_2_^49,52^, and/or in glass dissolution rates triggered by strong hydrological perturbations^20^. Possible other metastable precursors, such as lepidocrocite, green rusts, and hydrous Fe-oxide (i.e., Fe(OH)_2_) have not been identified by our analyses but should exist given the high rates of Cl^−^ and SO_4_^2−^ deposition from sea salt aerosols to the site^20^. Analogy from other studies indicates that these metastable products probably exist only as short-term, intermediate nanoparticles during ferrihydrite-OM coprecipitation^43,48,53,54^.

With respect to the mineralogical composition of rhyolitic MB_2_ tephra as the parent material, focusing solely on the alteration of the glass component would not be expedient. The bulk tephra contains 3.95 wt.% Fe_2_O_3_t, which is significantly higher than the Fe_2_O_3_t content of the matrix glass^20,39^ that was considered above. Abundant titanomagnetite phenocrysts in MB_2_ ash are least resistant to chemical weathering, while other Fe-bearing silicates (hornblende, clinopyroxene) remain mostly unaltered in these soils^20^. Our Rietveld analysis provides the mass balance for the iron budgets within MB_2_ tephra. Knowing its bulk composition and the average 1.4 wt.% Fe_2_O_3_t in matrix glass^26,39–41^ we estimate that the glass contains only ~ 25% of total Fe, whereas ~ 38% is contributed by titanomagnetite microcrysts containing 71.7 wt.% Fe_2_O_3_t on average (Figs. S3 and S4). Thus, the weathering of MB_2_ titanomagnetite crystals represents a substantial, but so far underappreciated contribution to the iron biogeochemistry in these Andosols.

Accordingly, recent research emphasizes the function of the mixed-valent Fe-oxide magnetite as highly effective natural battery in the redox-induced cycling of iron by microorganisms and the interaction with OL in soils and sediments, particularly under fluctuating water levels and the related redox changes^6,55^. This RAMP is principally introduced by the MB_2_ tephra to the regional soil systems in the form of nano- or micrometer-scale sized crystals (Figs. S3 and S4), but is no longer preserved intact, as XRD data^20^ and our Raman spectroscopy analyses indicate (Fig. 2). The Raman signals of mixed Fe-(hydr)oxides (Fig. 2c,d) as well as of pure hematite (Fig. 2e,f) from larger grains are presumably caused by specific Fe-(hydr)oxide (trans)formation processes restricted to MB_2_ titanomagnetite phenocryst alteration:

3 $${{Fe}^{2+}({Fe}^{3+},{Ti}^{4+})}_{2}{O}_{4}\stackrel{ +{O}_{2} }{\Longrightarrow }{ (Ti}_{0.5}^{4+}{\square}_{0.5}{)Fe}_{2}^{3+}{O}_{4}$$

In redox environments of soils and sediments, low-temperature oxidation of titanomagnetite is common and temporarily forms maghemite^50,56,57^ (or ‘titanomaghemite’ in Eq. 3). During this process of pseudomorphism (also called maghemitization), Fe^2+^ in magnetite is oxidized while the spinel lattice is left intact with one-sixth of the octahedral sites remaining vacant^58^. We consider Ti-bearing maghemite (Fig. 3) as the metastable, topotactic intermediate product of the low-temperature oxidation to hematite as the stable end product (Eq. 4):

4 $${{(Ti}_{0.5}^{4+}{\square}_{0.5}{)Fe}_{2}^{3+}{O}_{4}\stackrel{ +{O}_{2} ({+Fe}_{ dissolved}^{2+} +microbes) }{\Longrightarrow}{Fe}_{2}^{3+}{O}_{3}} (+{Ti}^{4+})$$

In addition, bacterially-mediated oxidation processes can ultimately enhance hematite formation from maghemite under acidic pH^59^—a process that is probably strongly promoted by the addition of aqueous Fe^2+^, donating electrons to hematite inducing crystal growth^52^. For this reason, maghemite substantially contributes to the total amount of hematite formed in these Andosols.

Similar (trans)formation pathways, comparable to those shown in Fig. 2, include the development of maghemitization rims around partially oxidized (titano)magnetite^60^. Maghemitization has also been observed in soils with andic properties^56^, can be mediated by abiotic and biotic processes simultaneously and is suggested to be amplified under varying environmental conditions under a humid climate^55^. It is noteworthy that, based on our Raman spectroscopy measurements (Fig. 3), we expect maghemitization to be more efficient for very fine-grained titanomagnetite, consistent with Yuan et al.^57^ and Qian et al.^60^. Thus, the majority of nano- to micrometer-sized titanomagnetite crystals within MB_2_ tephra should have already been transformed during the past ~ 4.2 kyrs. It follows from other sedimentary settings that intense maghemitization with subsequent Fe-(hydr)oxide precipitation can play an integral role in producing pronounced oxidation fronts in sapropels^60^, comparable to the process that resulted in elevated Fe_2_O_3_t contents at redox interfaces in these Ah-horizons described above (e.g., Table 1).

Irrespective of the topotactic oxidation of maghemite, another hematite formation pathway should be taken into account, if water-level-induced redox-fluctuations, the low pH, omnipresent OM decomposition and microbial activity are considered. An iron redox cycling in such a dynamic system typically includes various dissolution processes, such as reductive/oxidative dissolution^57,61^, dissolution by OL-complexation^6^ and the dissimilatory reduction by microbes^6,55,62^. Both, titanomagnetite as well as maghemite can efficiently be dissolved^63^, leading to a pronounced Fe^2+^ release from these minerals^54,57^. Hence, the dissolution of titanomagnetite (Ti-bearing maghemite, respectively) may also contribute to ferrihydrite precipitation via OM-rich, noncrystalline RAMPs as precursors (Eqs. 5–6), and thus, also to hematite production (Eq. 2). Etique et al.^62^ reported similar processes originating from the bioreduction of magnetite, including the precipitation of green rusts, siderite, ferrihydrite, and hematite.

5 $${{Fe}^{2+}({Fe}^{3+},{Ti}^{4+})}_{2}{O}_{4}\stackrel{{-Fe}_{ dissolved}^{2+}\stackrel{+OL +microbes}{\Longrightarrow }{ }_{noncyrstalline}RAMPs\stackrel{+{H}_{2}O +{O}_{2} +microbes}{\Longrightarrow } }{}{Fe}_{10}^{3+}{O}_{14}{(OH)}_{2} (+{Ti}^{4+})$$

6 $${(Ti}_{0.5}^{4+}{\square}_{0.5}{)Fe}_{2}^{3+}{O}_{4}\stackrel{{-Fe}_{ dissolved}^{2+}\stackrel{+OL +microbes}{\Longrightarrow }{ }_{noncrystalline}RAMPs\stackrel{+{H}_{2}O +{O}_{2} +microbes}{\Longrightarrow } }{ }{Fe}_{10}^{3+}{O}_{14}{(OH)}_{2} (+{Ti}^{4+})$$

Even though an enrichment of Fe-(hydr)oxides is reasonable at a redox interface, the fate of the potentially considerable amount of Ti^4+^ that is released during titanomagnetite (trans)formation (Eqs. 4–6) remains cryptic. We observe that iron and titanium synchronously accumulate in the uppermost soil column^20^. Therefore, we argue that (i) in the early stages of maghemitization the production of titanohematite is possibly favoured at an intermediate stage^56^, followed by the formation (ii) of nanoparticulate Ti-oxides such as pseudorutile (Fe^3+^_2_Ti^4+^_3_O_9_) and anatase (Ti^4+^O_2_) that precipitate from acidic solutions in OM-rich soils^64,65^. These Ti–rich nanoparticles are, however, likely disseminated and difficult to detect in the very fine-grained oxidation products.

**Figure 6:**
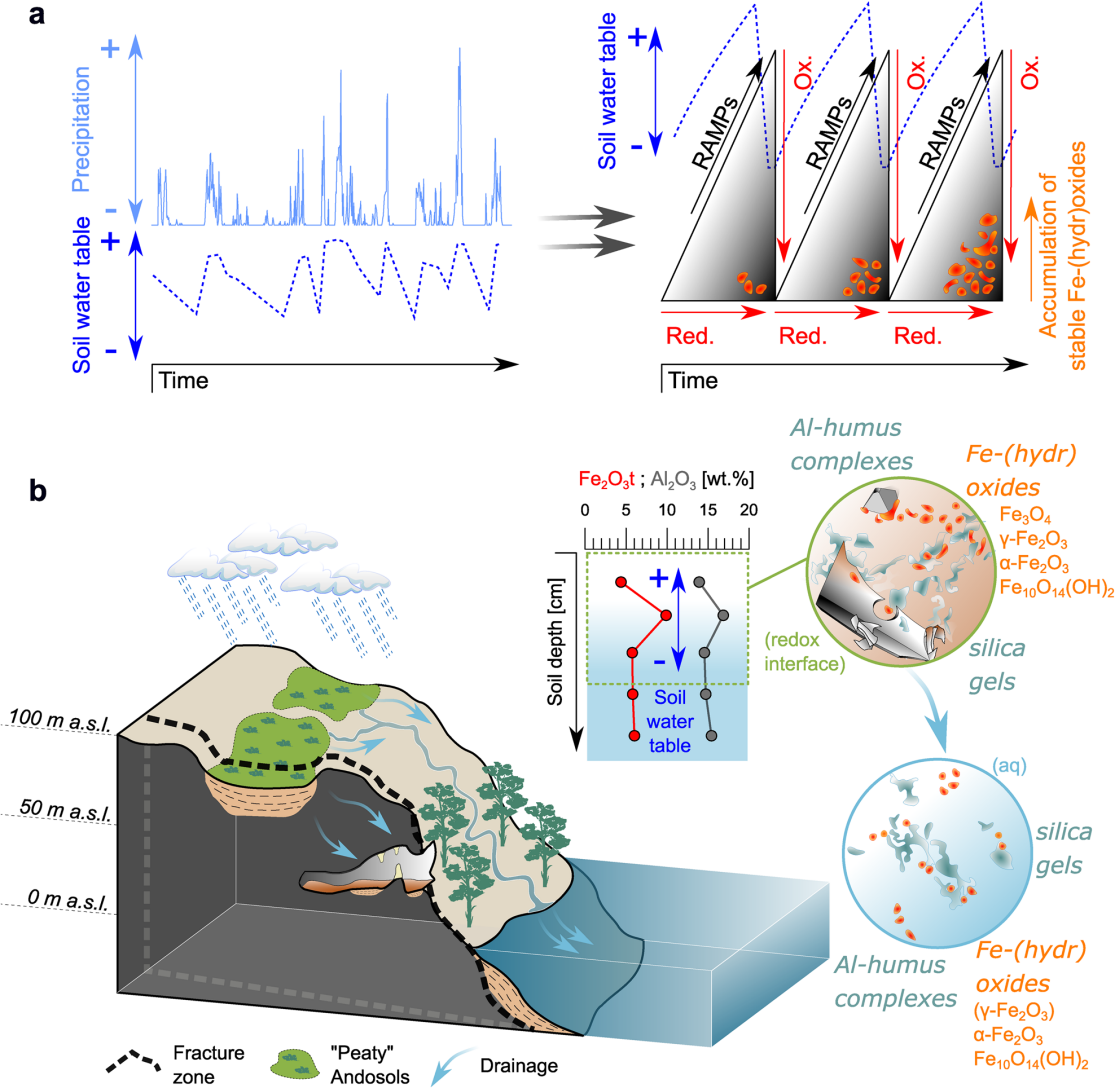
Synthesis of the controlling mechanisms on Fe-(hydr)oxide (trans)formation in and mobilization from peaty Andosols in this hyper-humid region. **(a**) The impact of varying rainfall intensity on soil water-level fluctuations is shown. Frequently recurring hydrological disturbances modulate the soil redox state (*Red*.  reducing conditions, *Ox.*  oxidizing conditions), affect iron redox cycling and lead to a continuous production of noncrystalline redox-active metastable phases (RAMPs) under reducing conditions and the precipitation of crystalline, thermodynamically stable Fe-(hydr)oxides by progressive, oxidative stages (after Peiffer et al.^23^, modified). (**b**) Schematic illustration of the study area (Klaes et al.^26^, modified) including suggested mobilization patterns of distinct Fe-(hydr)oxides accompanied by Al–humus complexes and amorphous silica originating from hydrological disturbance at the redox interface in these volcanic ash soils.

In summary, our data underline the ecological relevance of the element cycling in Patagonian Andosols, which is sensitively controlled by the complex interaction between these soils and the extreme and variable climate conditions. We suggest that both, abiotic as well as biotic terrestrial iron redox cycling are ultimately regulated and intensified by SWW-variations with important implications for the regional organic carbon (OC) budgets and micronutrient liberation. Climate as the main driver regulates differences in crystallinity, and thus, the bio-availability of the delivered Fe-phases^12,66,67^, strongly affecting runoff composition and, consequently, the marine primary productivity in adjacent fjords from seasonal to decadal time scales^18,19,68^. In this context, we assign particular importance to the continuous formation of noncrystalline RAMPs (Eqs. 1, 5 and 6), which predominantly precipitate during periods of higher rainfall (RAMP production in Fig. 6a) when oxidization to phases with higher crystallinity is inhibited^9,13,48,49^. Such noncrystalline, high-surface-area Fe-(hydr)oxides (e.g., ferrihydrite) stabilize coprecipitated OM and protect it from biodegradation under reducing conditions^12,42,43^. Unlike crystalline Fe-(hydr)oxides, these OM-associated noncrystalline Fe^2+/3+^-RAMPs and ferrihydrite coprecipitates should account to a great degree for terrestrial carbon sequestration^42,43^ and the provision of bio-available iron for fjord primary productivity^66,67^. In contrast, the precipitation of hematite would increase iron fixation at the soil redox inter-face. Such persistent retention of iron in soils through the physical accumulation of more thermodynamically stable hematite would occur only during phases of comparatively low rainfall, which will reduce the frequency and intensity of cycles of oxidative conditions in soils (Fig. 6a). At the same time, aerobic OM decomposition will be more pronounced^37,38^. Hematite (or partly maghemite) that may have formed this way would be released at rather high rates by frequent storm events after/during such less humid phases^21,69^ (Fig. 6b). Thus, it is likely that iron and OC exports occur mostly asynchronous and are determined by the frequency and duration of redox cycles in these Andosols or induced by extreme weather events. The bulk composition of the colloidal export beyond iron and OC may also vary with different proportions of Al-humus complexes and amorphous silica (Fig. 6b), in particular during drastic environmental perturbations, such as the millennium-scale acidification phase following the MB_2_ eruption^24^.

These peaty volcanic ash soils play a fundamental role in the Late Holocene biogeochemical cycling within the remote South Patagonian fjord ecosystems, because Andosol-sourced micronutrient fluxes potentially are of similar importance as those from glacial erosion^18,19,66,67^. Therefore, the successful identification of distinct Fe-(hydr)oxide (trans)formation pathways will enable a more precise evaluation of ecosystem properties and interpretations deduced from regional paleoenvironmental archives. For instance, typically used Fe-(hydr)oxide proxies in sedimentary records (goethite/hematite ratios^3,50^) are not applicable under the climatic controls described here. Evidence for the long-term (paleo)environmental importance of this Andosol-sourced micronutrient supply exceeding 4 kyrs is provided by the tephra-sourced Fe-(hydr)oxides preserved in laminae of stalagmite MA1^26^, which grew in a cave underneath these soils (Fig. 6b). NanoSIMS measurements along MA1 clearly reflect the spatial distribution of ^28^Si^−^, ^16^O^−^, ^56^Fe^16^O^−^, ^27^Al^16^O^−^, ^32^S^−^, ^12^C^−^ and ^12^C^14^N^−^ that we observe in the soil aggregates (Figs. 5 and S7). However, in addition to the iron leached from volcanic glass, the continous release of bio-available Fe from titanomagnetite phenocrysts represents a further, so far underappreciated but potentially important source for the prolonged land-to-fjord micronutrient supply from these Andosols. Such enhanced flux of bio-available Fe and other essential micronutrients from tephra may outlast the previously estimated ~ 6 kyrs of environmental impact after the deposition of the MB_2_ ash layer^20^. This ‘tephra-effect’ may also surpass the seasonally limited inputs from glacial meltwater in this region^69^.

## Methods

### Sampling and sample preparation

The samples from the MA catchment were collected during the 2015 austral winter field campaign with R/V Gran Campo II described in Klaes et al.^20^. One Ah-horizon of the MA catchment was sampled at five different depths (0–4, 4–11, 11–15, 15–19 and 19–22 cm). The sampling position of this Ah-horizon was located exactly in the middle of the catchment. Close agreement between the compositions of the topmost sample in our study with data from four Ah-horizons of the same area^20^ confirmed the representativeness of the selected site. Individual soil aggregates of ~ 2 to 10 mm size (Fig. 1a) were collected from 5 to 15 cm depth of the Ah-horizons of four Andosol profiles. Tephra pumice samples from the MB_2_ layer at 35 to 42 cm depth were taken. Furthermore, eleven further samples from 0 to 5 cm depth were collected from evenly distributed sites in the catchment.

Bulk soil subsamples analyzed in the present study were shock-frozen with liquid nitrogen and air-dried under vacuum for 48 h, sieved to a grain size < 2 mm and ground to powders by an agate ball mill. Soil aggregates and pumice samples were carefully cleaned with H_2_O_dest_ and air-dried. Then, 50 selected soil aggregates and 10 g pumice of each tephra layer were placed on glass slides (example given in Fig. 1a) and embedded in Buehler EpoThin epoxy resin under vacuum for 72 h. The resin blocks with the incorporated specimens were cut and polished. Weathered granite fragments found in the Cr-horizons of these soils (~ 40 cm soil depth^20^) were used to produce five polished, 30 µm thick thin sections to study their alteration rims.

### Bulk geochemistry and selective extractions of pedogenic (hydr)oxides

The bulk composition of the Ah-horizon samples from the middle of the MA catchment (major elements and concentrations of Rb and Sr) was determined on powdered specimens using a sequential X-ray fluorescence (XRF) spectrometer with a rhodium target X-ray tube (PANalytical AXIOS-Advanced) at the GZG (University of Göttingen). Analyses were performed on glass discs prepared by fully automated fusion at 1050 °C using a flux of ultra-pure LiBO_2_ and Li_2_B_4_O_7_. A wavelength-dispersive standard calibration routine with international reference materials (34 major and trace elements) was set up. During the measurements, peak overlap interferences were avoided by the use of suitable diffraction crystals, line overlap interferences have been corrected by the PANalytical software package SuperQ 4. The analytical precision for major elements was better than 2%. For trace elements, 1σ standard deviations were in the range of 2 to 5% at concentration levels of 30 to 10 ppm (detection limits varied between 5 and 0.1 ppm). Pedogenic (hydr)oxide concentrations of Fe and Al of the eleven samples from 0 to 5 cm depth were measured at the Soil Science Department of Trier University, targeting noncrystalline and crystalline phases. According to the recommendation by Rennert^70^, powdered samples were used. The measurements were conducted after ammonium oxalate extraction (Fe_o_, Al_o_; noncrystalline) at pH 3.0^71^ and the citrate bicarbonate dithionite extraction procedure (Fe_d_, Al_d_; noncrystalline and crystalline) after Mehra and Jackson^72^. A Varian AA240FS fast sequential atomic absorption spectrometer was used coupled to a Varian PSD120 sample dispenser to perform duplicate analyses of each sample, yielding standard errors smaller than 5% for all measurements. Blank values served for correction. Detection limits for the measured Fe and Al concentrations were in sub-ppm range^73^.

### Relative phenocryst abundances in MB_2_ tephra

The relative proportions of abundant phenocrysts in pristine MB_2_ tephra were calculated from the XRD data presented in Klaes et al.^20^. Match! software (version 3.13) was used in combination with the Rietveld analysis tool implemented in the FullProf Suite^74,75^ (version 7.40). For phase identification, the quartz peak at 3.342 Å (Cu-Kα radiation) was applied as internal standard. The accuracy of the Rietveld analysis (p = 0.05; χ^2^ = 9.5; weighted average Bragg factor *R*_wp_ = 81.9%) was accepted as good, following the recommendations of Toby^76^. The result from the Rietveld analyses was set to a matrix glass-to-phenocrysts relationship of 70:30^20^ (cristobalite XRD pattern served as glass component) after normalization to 100%. Minor amounts of quartz and pyrite were excluded from the calculation due to detrital contamination from surrounding bedrock lithologies and sulfide formation in the suboxic subsoil^20^. The plausibility of this so-calculated phenocryst composition was ensured by accompanying optical microscopy, consistency with the published plagioclase content in MB_2_ tephra (15–20 vol.%^77^) and by checking the modelled composition against the bulk chemistry of MB_2_ ash^20,39^. We accepted a deviation of ± 5% for SiO_2_, Al_2_O_3_, TiO_2_ and Fe_2_O_3_t. For individual mineral phases of the final phenocryst composition, the assumed relative error was better than ± 5%.

### Scanning electron microscopy

Scanning electron microscopy was carried out with a LEO 435VP at the Geology Department of Trier University and a Carl Zeiss MERLIN VP compact at the IOW, Warnemünde. In both cases, samples were vacuum sputter-coated with gold and an acceleration voltage of 15 kV was applied to produce high-resolution images in back-scattered electron (BSE) mode. At the IOW, EDS analyses (point analyses, area measurements and element mappings) were performed with an Oxford Instruments AztecEnergy system equipped with a X-MAX^N^80 SDD detector. Elements were detected on the Kα line with a spatial resolution of 1 nm and an energy range of 20 keV. The residence time for each pixel of the BSE images was set to 60 ms for element mappings/area measurements and to 15 ms for single-point analyses, respectively. The EDS measurements were calibrated with various natural and synthetic standards. At concentrations < 1 wt.%, the quantification via EDS yields an increasing relative error (> 70%^78^). Therefore, analyses with concentrations < 1 wt.% were discarded.

### Raman imaging spectroscopy

The AOIs selected for investigations with Raman spectroscopy on resin-embedded soil aggregates focused on distinct Fe-(hydr)oxide crusts and crystals > 2 μm to increase the accuracy of spectrum acquisition and to reduce interference signals, i.e., caused by fluorescence emitted by OM^79^. On thin sections, we analyzed secondary Fe-phases formed in fissures and alteration rims of weathered granite for comparison.

For the identification of Fe-(hydr)oxides, a WITec alpha300 R+ confocal Raman-imaging microscope system at the Soil Science Department of Trier University was used—equipped with a frequency-doubled Nd:YAG laser at λ = 532 nm (WITec UHTS300s_Green_NIR). The microscope was coupled to a WITec UHTS 300 VIS–NIR spectrograph (cooled down to − 60 °C) and a CCD camera for detection with 2000 × 256 pixels. Spectral resolution was ~ 2.5 cm^−1^ (with diffraction grating of 600 grooves mm^−1^). The laser beam was focused using a 100 × objective magnitude (Carl Zeiss EC Epiplan-Neofluar Dic 100×/0.9), resulting in a lateral resolution < 1.0 μm. To avoid a thermal degradation of Fe-(hydr)oxides, e.g., the dehydration of goethite and transformation to hematite, laser power should not exceed 1.0 mW^28,29^. Therefore, laser powers applied in this study varied between 0.1 and 1.0 mW (Table S1). We preferentially chose to obtain several spectra with short acquisition times from the same AOI to reduce background fluorescence, interference signals from cosmic radiation^79^ and to inhibit degradation effects. The configuration of these single point measurements were adjusted specifically with respect to the laser power used and the occurring mineral phase (Table S2). Before and after each measurement, the AOIs were inspected by white light illumination microscopy in order to detect any possible laser-induced degradation. Accordingly, the measurements on goethite (granite thin sections; Fig. S5) confirmed that the chosen configuration was suitable for Fe-(hydr)oxide identification and had not caused thermal degradation of the samples. Raman spectra were referenced using the 521 cm^−1^ band of a silicon wafer and evaluated (accumulation, baseline correction, peak fitting, clustering) with the WITec control FIVE software.

### NanoSIMS

The 27 AOIs for NanoSIMS investigations were chosen with respect to the characteristic proportions of OM, residual volcanic glass and other silicate components in the soil aggregates as indicated by the prior documentation with optical microscopy and SEM-BSE imagery (Figs. 1a and S8). The use of NanoSIMS is in particular suitable for gathering (sub)micrometer-scale information of the distribution of Fe-and Al-(hydr)oxides in the matrix of the soil aggregates, because it allows the discrimination between Fe and Al in silicate- and non-silicate phases, i.e., (hydr)oxides^80–82^. This technique only reflects relative element concentrations of the sample due to differences in ionization potential^83^, which could be largely influenced by crystallinity. In areas, where high polyatomic secondary ion (e.g., ^27^Al^16^O^−^, and ^56^Fe^16^O^−^) counts were measured, NanoSIMS documents phases rich in Al or Fe but also O, and thus, emphasizes abundant Fe and Al in (hydr)oxides in contrast to better crystalline silicates^12,80^.

The NanoSIMS measurements were carried out at the Chair of Soil Science of the Technical University of Munich. A Cameca NanoSIMS 50L was used to explore the AOIs on polished samples with a Cs^+^ primary ion beam (impact energy of 16 keV) after coating with Au/Pd layer (ca. 30 nm, Polaron Emitech SC7640) to account for charging during the measurements. An additional compensation of charging was guaranteed by the use of the electron flood gun of the NanoSIMS. A high primary beam current was applied to sputter away impurities and the Au/Pd coating, and to implant the Cs^+^ ions into the sample, while secondary ion yields increased until reaching a steady state. Scanning with the focused primary beam (ca. 2 pA) resulted in a lateral resolution of ca. 120 nm. For the collection of ^12^C^−^, ^16^O^−^, ^12^C^14^N^−^, ^28^Si^−^, ^32^S^−^, ^27^Al^16^O^−^, and ^56^Fe^16^O^−^ secondary ions, electronic dead time was fixed at 44 ns. For an accurate mass resolution/ mass isobar separation, D1_3, ES_3 and AS_2 slits and apertures were used. The recording of secondary ions on a 30 × 30 μm field of view (256 × 256 pixels) was performed with a dwell time of 1 ms pixel^−1^ and 30 planes per scan. The evaluation of NanoSIMS data (dead time and drift correction and accumulation of single planes as well as the calculation of mass ratios and composite images) was done using ImageJ^84^ combined with the Open-MIMS plugin^85^.

The spatial distribution of intra-aggregate OM was distinguished from voids filled with the used epoxy resin by optical microscopy and calculated mass ratios/composite images (Fig. S8). Reference measurements on pure Buehler EpoThin revealed that its composition is characterized by a considerably low amount of ^12^C^14^N^−^ secondary ion counts in contrast to high ^12^C^−^. Therefore, the mass ratios/composite images allow a clear identification of the resin in our samples by its specific ^12^C^−^ signature compared to OM components that are rich in ^12^C^14^N^−^^81–83^. In addition, Figs. S9 and S10 demonstrate that the used resin does not contain notable trace concentrations of neither N, Si, S, Al nor Fe, which could potentially cause interferences with secondary ion counts measured by NanoSIMS. Therefore, a contamination of our samples by the preparation process and used adhesives can be ruled out.

### Statistical analyses

Statistical analyses shown in Figs. 1b and S2 were calculated using the ‘analysis tool’ implemented in Microsoft Excel. The statistical significance of the calculated linear and logarithmic regression models was determined on a significance level of p = 0.05. The indicated confidence intervals express 95% levels.

## Supplementary Information

Supplementary Information.

## Data Availability

The datasets generated during the current study can be obtained from the Zenodo Repository (https://doi.org/10.5281/zenodo.7528253).
