# Supplementary material for: Iron (hydr)oxide formation in Andosols under extreme climate conditions
Source: Sci Rep. 2023 Feb 16;13:2818. doi: 10.1038/s41598-023-29727-1 (PMC9935883; doi:10.1038/s41598-023-29727-1)
Supplement: Supplementary file 1 — Supplementary Information. [file 41598_2023_29727_MOESM1_ESM.pdf]

Supplementary Information

Article title: Iron (hydr)oxide formation in Andosols under extreme climate conditions

Authors: Björn Klaes[1,2*], Sören Thiele-Bruhn[2], Gerhard Wörner[3], Carmen Höschen[4], Carsten W. Mueller[4,5], Philipp Marx[2], Helge Wolfgang Arz[6], Sonja Breuer[7], Rolf Kilian[1,8†]

[1]Geology Department, Trier University, Campus II (Geozentrum), Behringstraße 21, 54296 Trier, Germany; klaesb@uni-trier.de

[2]Soil Science Department, Trier University, Campus II (Geozentrum), Behringstraße 21, 54296 Trier, Germany; thiele@uni-trier.de, phipmarx@kabelmail.de

[3]Division of Geochemistry and Isotope Geology, GZG, Georg-August-University Göttingen, Goldschmidtstraße 1, 37077 Göttingen, Germany; gwoerne@gwdg.de

[4]Soil Science, Research Department Life Science Systems, TUM School of Life Sciences, Technical University of Munich, Emil-Ramann-Straße 2, 85354 Freising-Weihenstephan, Germany; carmen.hoeschen@tum.de

[5]Department for Geosciences and Environmental Management, University of Copenhagen, Øster Voldgade 10, 1350 København K, Denmark; cm@ign.ku.dk

[6]Marine Geology Section, Leibniz Institute for Baltic Sea Research Warnemünde (IOW), Seestraße 15, 18119 Rostock, Germany; helge.arz@io-warnemuende.de

[7]Federal Institute for Geosciences and Natural Resources (BGR), Stilleweg 2, 30655 Hannover, Germany; sonja.breuer@bgr.de

[8]University of Magallanes, Avenida Bulnes 01855, Punta Arenas, Chile

* = corresponding author (B. Klaes);

† = deceased

Table S1: Compilation of published wavenumbers of diagnostic bands of secondary Fe-minerals used for phase identification with Raman spectroscopy in this study.

Mineral phase	Diagnostic Raman bands [cm^{-1}]														Reference
Goethite		244	299	385		480	548		681						*Hanesch[1]*
		243	299	385		479	550		685	993	1120	1255			*de Faria et al.[2]*
	205	247	300	386	418	481	549								*Oh et al.[3] and references therein*
		245	300	390	420	480	550		685						*Oh et al.[3] and references therein*
		248	303	397		485	554		680	1002	1120				*Oh et al.[3] and references therein*
		245	300	390		485	550								*Oh et al.[3] and references therein*
	205	245	300	390	415	480	550		685	1005	1300				*Bridges et a[4]*
	162	243	297	384		477	545		655						*Das and Hendry[5]*
			298	397	414	474	550								*Thibeau et al.[6]*
		225	297	393		482	565		676						*Legodi and de Waal[7]*
		243	297	392	477	546		610	682	993	1113	1209	1292		*Froment et al.[8] and references therein*
		243	299	385	479	550			685	993	1120	1255			*Froment et al.[8] and references therein*
Hematite	225		245	290-300	412										*Hanesch[1]*
	219-226		236-245	282-292; 295-299	395-410	492-497	596-612								*de Faria et al.[2]*
	225		245	291-292	411	497-500	611-612					1321			*Oh et al.[3]*
	225		245	290	405	495	605					1310			*Bridges et al[4]*
	222	230	290		408	490	607								*Das and Hendry[5]*
	227		245	293-298	414	501	612								*Thibeau et al.[6]*
	223		245	291	407	495	608								*Legodi and de Waal[7]*
	224		243	290	406	494	607	655	813	1055	1100	1310			*Froment et al.[8] and references therein*
	225		247	293	412	498	613					1320			*Froment et al.[8] and references therein*
	225		247	292	411	496	610					1318			*Chourpa et al.[9]*
Maghemite			350			512			664-665	726-730					*Hanesch[3]; Jacintho et al.[10]*
			350			500				700		1320		1560	*de Faria et al.[2]; Mazzetti and Thistlethwaite[11]*
				381	486				670	718					*Oh et al.[3] and references therein*
	265	300	345	395		515		645	670	715			1440		*Oh et al.[3] and references therein*
			350			505			660	710			1425		*Oh et al.[3] and references therein*
				380	460	510			670	720	1160		1400		*Bridges et al[6]*
	290	339		378	479	507	575	630	657	723	1172		1417		*Froment et al.[8]*
		330		380	482	502			652	703					*Chourpa et al.[9]*
Magnetite				310					540; (532-530)			670; (661-676)			*Hanesch[1]*
				302				513	534			663			*de Faria et al.[2] and references therein*
											616	663			*de Faria et al.[2] and references therein*
										550		670			*de Faria et al.[2] and references therein*
				298	320	420				550		676			*de Faria et al.[2] and references therein*
				300	320	420				560		680			*de Faria et al.[2] and references therein*
				298	319	418				550		676	1322		*de Faria et al.[2] and references therein*
									540			665			*de Faria et al.[2] and references therein*
										550		670			*de Faria et al.[2] and references therein*
									532			667			*Oh et al.[3] and references therein*
											616	663			*Oh et al.[3] and references therein*
				298	319	418				550		676	1322		*Oh et al.[3] and references therein*

				295				521		662			*Bridges et al*[4]
								536		667	1337		*Das and Hendry*[5]
									616	663			*Thibeau et al.*[6]
				307				532		667			*Legodi and de Waal*[7]
				292				524		665			*Froment et al.*[8] *and references therein*
				300				532		661			*Froment et al.*[8] *and references therein*
	194	258	276	303	327	456	508	528		662			*Chourpa et al.*[9]
Siderite	184		287							731	1090		*Hanesch*[1]
	180		282							722	1082		*Das and Hendry*[5]
Ferrihydrite					370		510			710			*Hanesch*[1]
					361		508			707	1045		*Das and Hendry*[5]
					370		510			710		1340	*Mazzetti and Thistlethwaite*[11]
					358		513		676	722	1046	1377	*Müller et al.*[12]

Table S2: Applied laser powers and configuration (accumulated quantity x duration of individual measurements) of the analyses performed with Raman spectroscopy. Abbreviations: Goe – goethite; Hem – hematite; Mgn – magnetite; Mgh – maghemite; Sid – siderite.

	Granite thin sections			Soil aggregates		
Mineral phase	Laser power [mW]	Configuration/ acquisition time	Figure	Laser power [mW]	Configuration/ acquisition time	Figure
Goe	0.7	20 × 2 s	Fig. S5	-	-	-
	0.7	20 × 0.5 s	Fig. S5	-	-	-
	1	10 × 0.5 s	Fig. S5	-	-	-
Hem-Mgn-Mgh	-	-	-	0.7	20 × 2 s	Fig. 2
	-	-	-	1	10 × 5 s	Fig. 2
Mgh	-	1	-	0.1	20 × 2 s	Fig. 3
	-	0.5	-	0.1	20 × 2 s	Fig. 3
	-	0.2	-	0.1	20 × 5 s	Fig. 3
	-	1	-	0.1	1 × 60 s	Fig. 3
	-	-	-	0.1	20 × 5 s	Fig. 3
Hem	0.7	20 × 0.5 s	Fig. S5	1	20 × 5 s	Fig. 4
	-	-	-	0.5	20 × 5 s	Fig. 4
	-	-	-	0.2	10 × 10 s	Fig. 4
	-	-	-	1	10 × 5 s	Fig. 4
Sid				1	1 × 300 s	Fig. S6

Fig. S1:

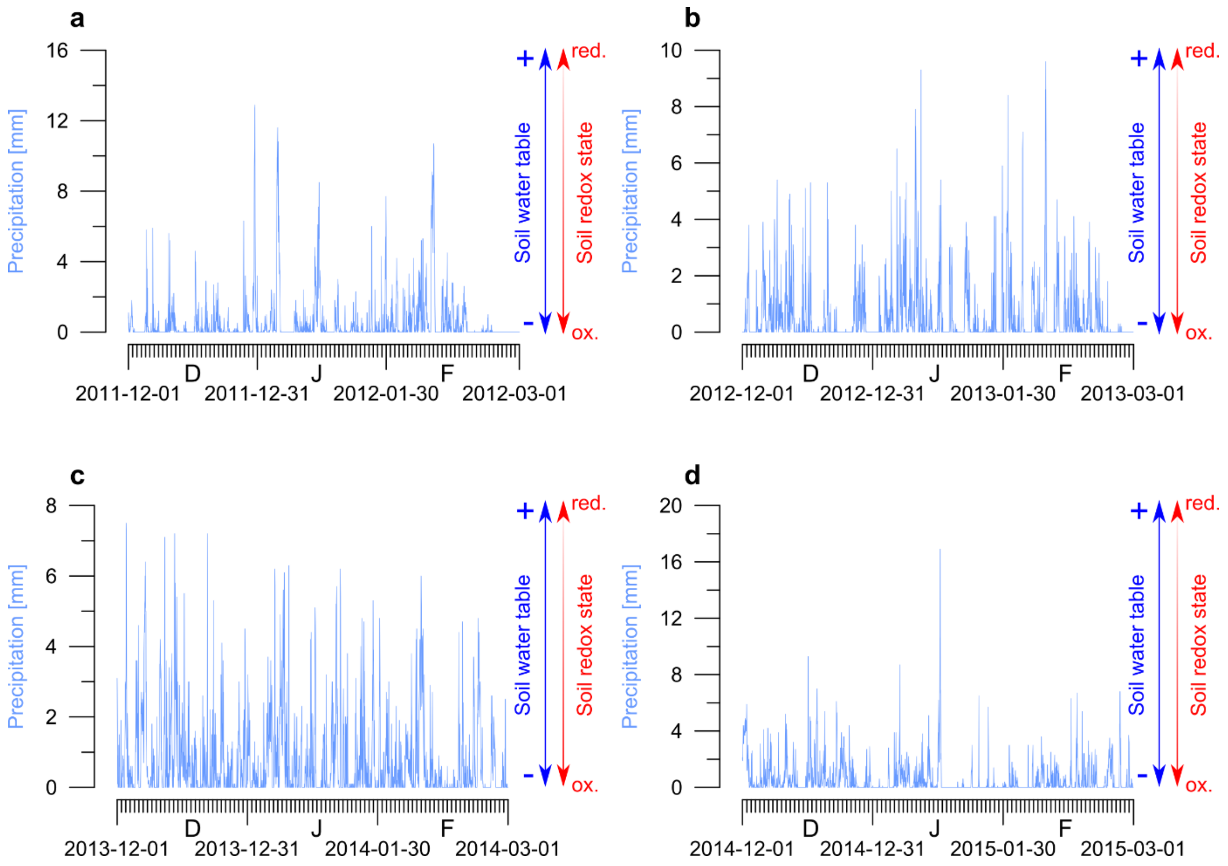

Variations in daily precipitation during austral summer recorded by automatic weather station Arévalo[13]. December, January and February (DJF) precipitation data are displayed for the years **a** 2011/2012, **b** 2012/2013, **c** 2013/2014 and **d** 2014/2015. The rainfall-driven control on soil water-level fluctuations, and thus, the suggested changes in the soil redox state[14], is indicated.

Fig. S2:

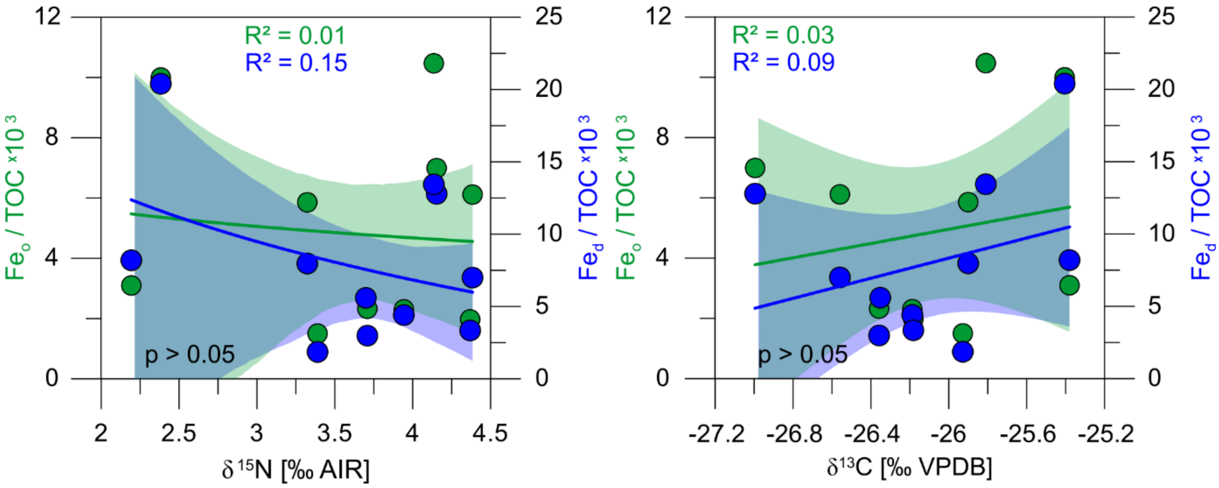

Wet-chemical extractions of iron from topsoils and their relation to organic matter turnover. Fe_o and Fe_d concentrations plotted versus $\delta^{13}C$ and $\delta^{15}N$ values indicate the relationships between noncrystalline/total Fe-(hydr)oxide content and organic matter turnover. Subscripts refer to the treatments used for wet-chemical extraction (citrate bicarbonate dithionite – Fe_d, ammonium oxalate – Fe_o). Shaded fields represent 95% confidence intervals.

Fig. S3:

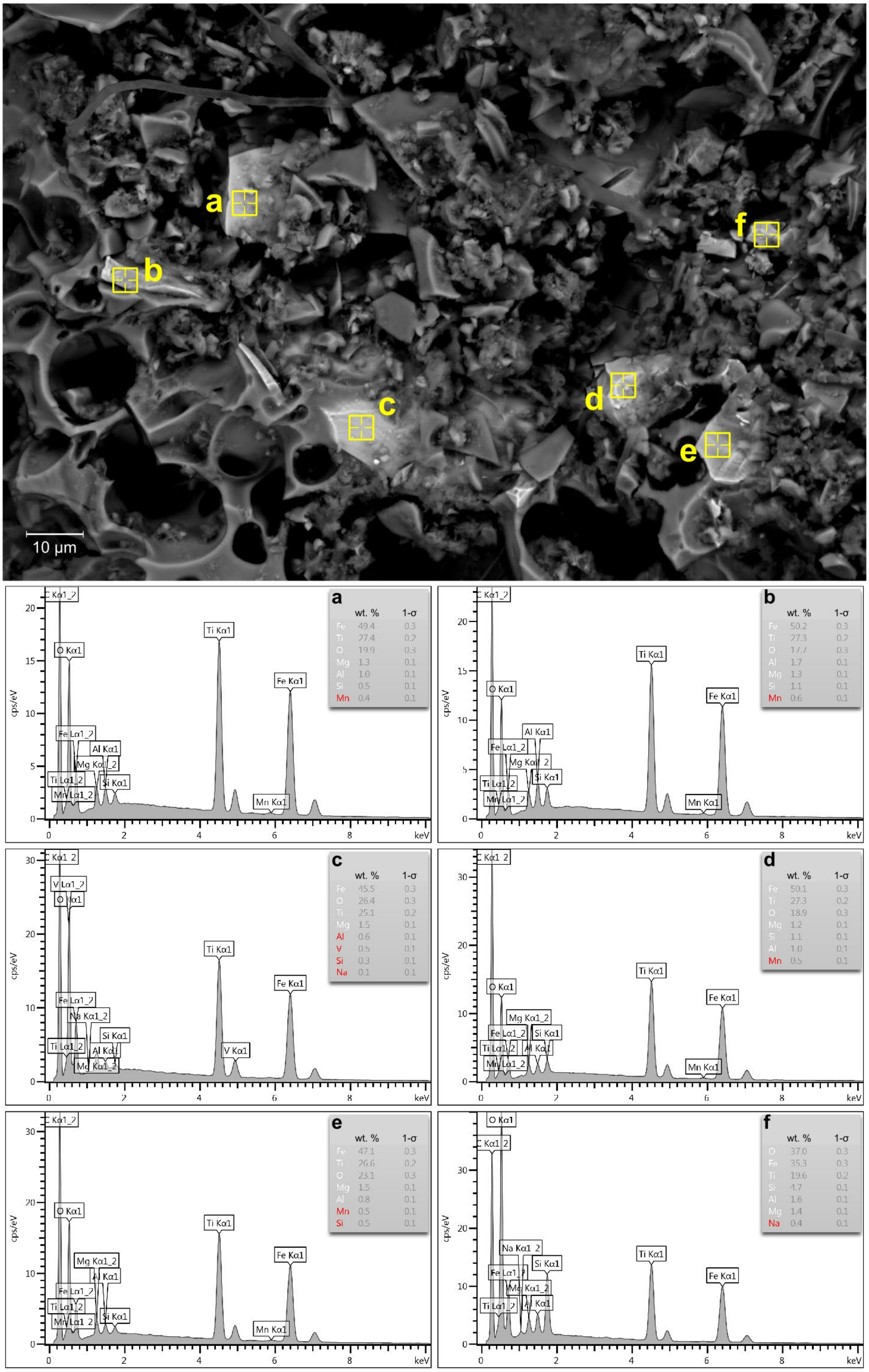

a
b
c
d
e
f
10 µm
a
wt. % 1-σ
Fe 49.4 0.3
Ti 27.4 0.2
O 19.9 0.3
Mg 1.3 0.1
Al 1.0 0.1
Si 0.5 0.1
Mn 0.4 0.1
b
wt. % 1-σ
Fe 50.2 0.3
Ti 27.3 0.2
O 17.7 0.3
Al 1.7 0.1
Mg 1.3 0.1
Si 1.1 0.1
Mn 0.6 0.1
c
wt. % 1-σ
Fe 45.5 0.3
O 26.4 0.3
Ti 25.1 0.2
Mg 1.5 0.1
Al 0.6 0.1
V 0.5 0.1
Si 0.3 0.1
Na 0.1 0.1
d
wt. % 1-σ
Fe 50.1 0.3
Ti 27.3 0.2
O 18.9 0.3
Mg 1.2 0.1
Si 1.1 0.1
Al 1.0 0.1
Mn 0.5 0.1
e
wt. % 1-σ
Fe 47.1 0.3
Ti 26.6 0.2
O 23.1 0.3
Mg 1.5 0.1
Al 0.8 0.1
Mn 0.5 0.1
Si 0.5 0.1
f
wt. % 1-σ
O 37.0 0.3
Fe 35.3 0.3
Ti 19.6 0.2
Si 4.7 0.1
Al 1.6 0.1
Mg 1.4 0.1
Na 0.4 0.1
cps/eV
keV
C Kα1_2
O Kα1
Ti Kα1
Fe Kα1
Mn Kα1
Fe Lα1_2
Al Kα1
Mg Kα1_2
Si Kα1
Ti Lα1_2
Mn Lα1_2
V Lα1_2
Na Kα1_2
V Kα1

Chemical compositions of titanomagnetite phenocrysts in MB_2 tephra. SEM micrograph showing an excerpt of a pumice particle from an MB_2 tephra layer and SEM-EDS single-point measurements of titanomagnetite phenocrysts (**a-f**). Yellow markings indicate the localizations of the measurements.

Fig. S4:

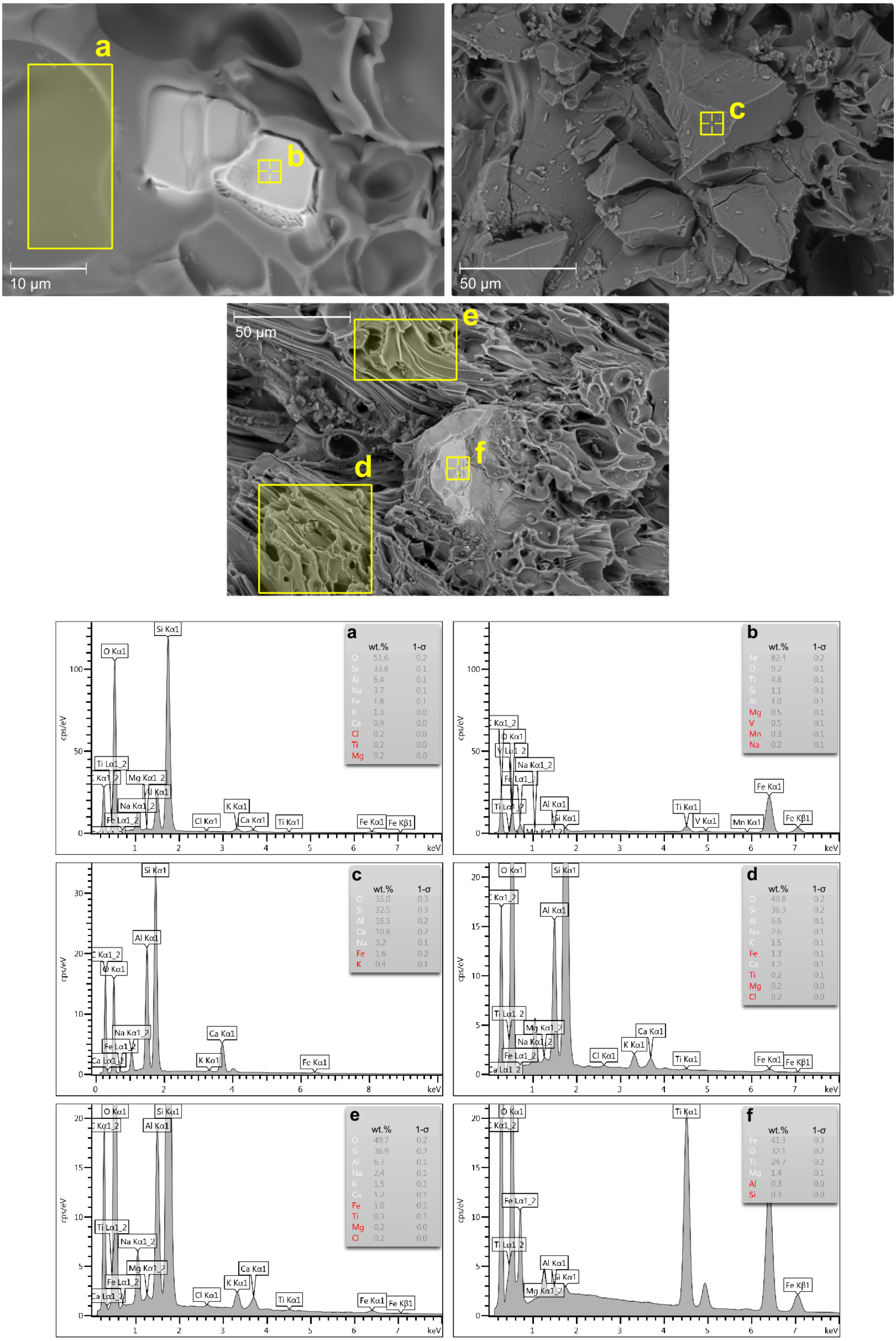

a
b
10 µm
c
50 µm
e
50 µm
d
f
cps/eV
keV
a
wt.% 1-σ
O 51.6 0.2
Si 33.8 0.1
Al 6.4 0.1
Na 3.7 0.1
Fe 1.8 0.1
K 1.3 0.0
Ca 0.9 0.0
Cl 0.2 0.0
Ti 0.2 0.0
Mg 0.2 0.0
b
wt.% 1-σ
Fe 82.4 0.2
O 9.2 0.1
Ti 4.8 0.1
Si 1.1 0.1
Al 1.0 0.1
Mg 0.5 0.1
V 0.5 0.1
Mn 0.3 0.1
Na 0.2 0.1
c
wt.% 1-σ
O 35.0 0.3
Si 32.5 0.3
Al 16.5 0.2
Ca 10.8 0.2
Na 3.2 0.1
Fe 1.6 0.2
K 0.4 0.1
d
wt.% 1-σ
O 49.8 0.2
Si 36.3 0.2
Al 6.6 0.1
Na 2.6 0.1
K 1.5 0.1
Fe 1.3 0.1
Ca 1.2 0.1
Ti 0.2 0.1
Mg 0.2 0.0
Cl 0.2 0.0
e
wt.% 1-σ
O 49.7 0.2
Si 36.9 0.2
Al 6.7 0.1
Na 2.4 0.1
K 1.5 0.1
Ca 1.2 0.1
Fe 1.0 0.1
Ti 0.3 0.1
Mg 0.2 0.0
Cl 0.2 0.0
f
wt.% 1-σ
Fe 41.3 0.3
O 32.1 0.2
Ti 24.7 0.2
Mg 1.4 0.1
Al 0.3 0.0
Si 0.3 0.0

Chemical compositions of MB_2 glass and embedded titanomagnetite phenocrysts. SEM micrographs showing excerpts of pumice particles from an MB_2 tephra layer. The yellow points and areas highlight the localizations of the measurements. SEM-EDS single-point and area measurements on volcanic glass (**a,c-e**) and titanomagnetite phenocrysts (**b,f**).

Fig. S5:

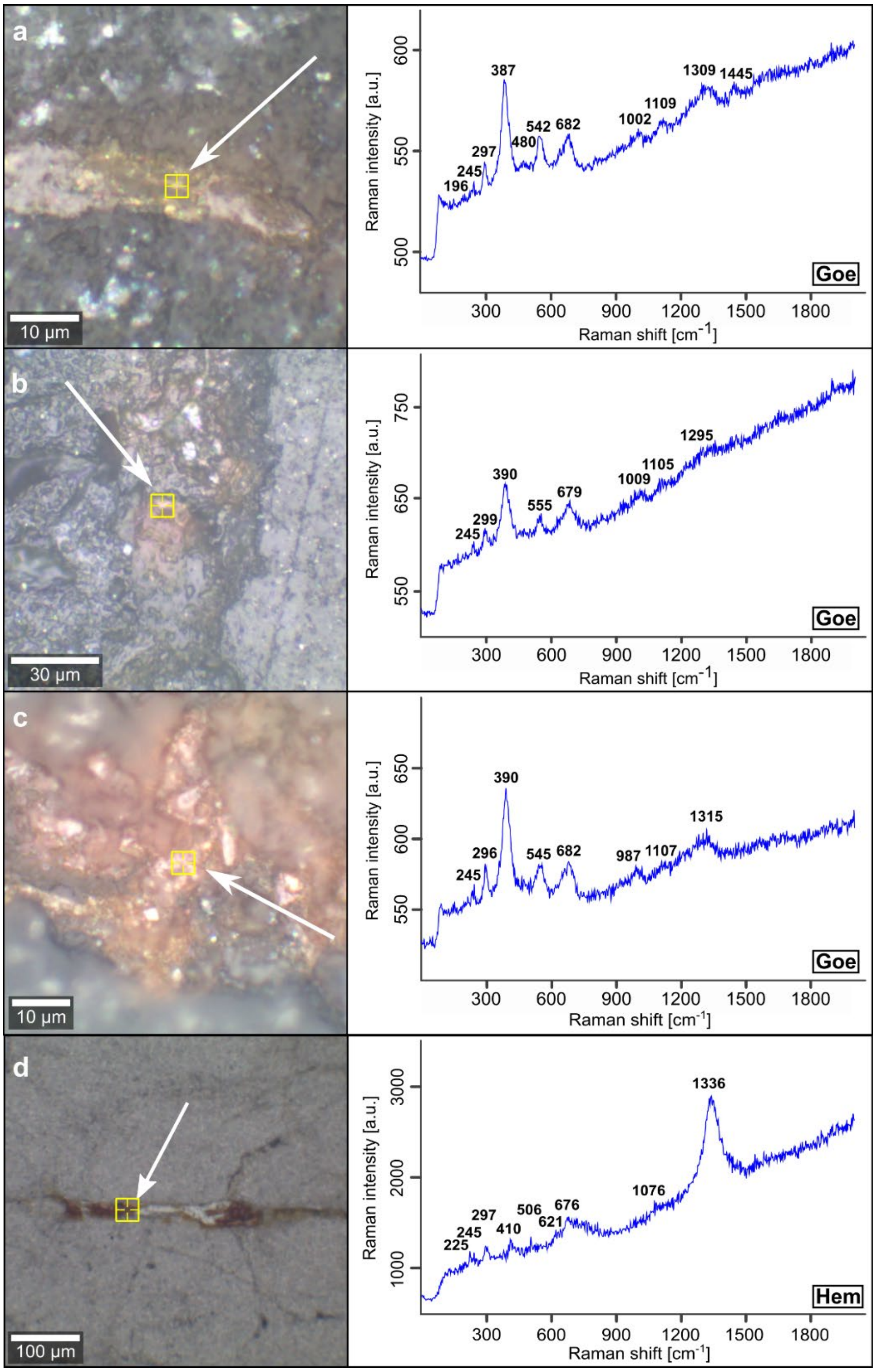

Raman spectra of hematite and goethite deduced from alteration rims of weathered granite. **a-d** Reflected light microphotographs in combination with Raman spectra of hematite and goethite. Bold numbers represent the wavenumbers of diagnostic Raman bands. Yellow markings indicate the localizations of the respective measurements. Goethite precipitates have been observed in cracks forming single crystals to crusts, depending on crack-width (**a-c**). Hematite fillings covering an extended crack network (**d**).

Fig. S6:

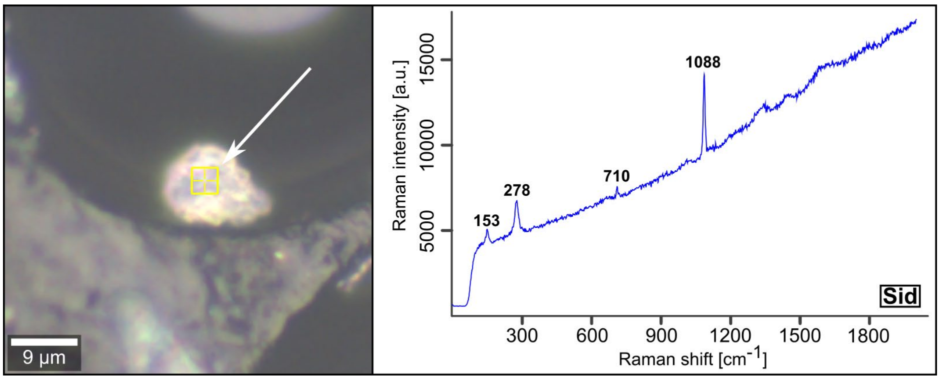

Raman spectra of siderite deduced from micrometer-scale crystals embedded in soil aggregates. Reflected light microphotograph combined with a Raman spectrum of siderite obtained from a crystal situated next to silicate glass. Bold numbers represent the wavenumbers of diagnostic Raman bands. The localization of the measurement is marked in yellow.

Fig. S7:

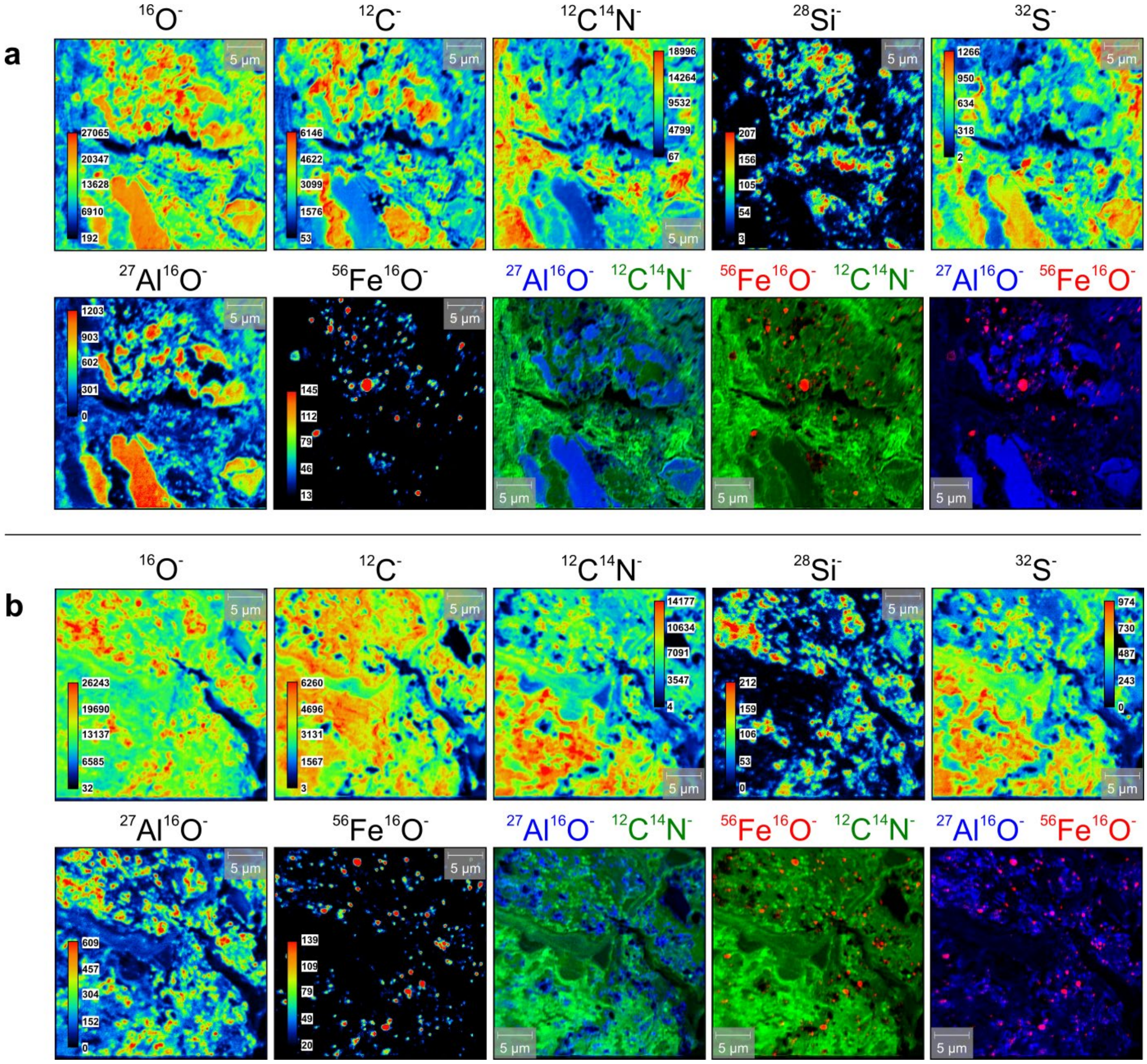

Additional NanoSIMS secondary ion mappings of to micrometer-scale intra-aggregate components. a,b Spatial distribution of $^{16}O^-$, $^{12}C^-$, $^{12}C^{14}N^-$, $^{28}Si^-$, $^{32}S^-$, $^{27}Al^{16}O^-$, and $^{56}Fe^{16}O^-$ secondary ions measured on 30 × 30 µm surfaces within the organic-rich matrix of the soil aggregates. In addition, composite images calculated from $^{12}C^{14}N^-$, $^{27}Al^{16}O^-$ and $^{56}Fe^{16}O^-$ secondary ion data are shown.

Fig. S8:

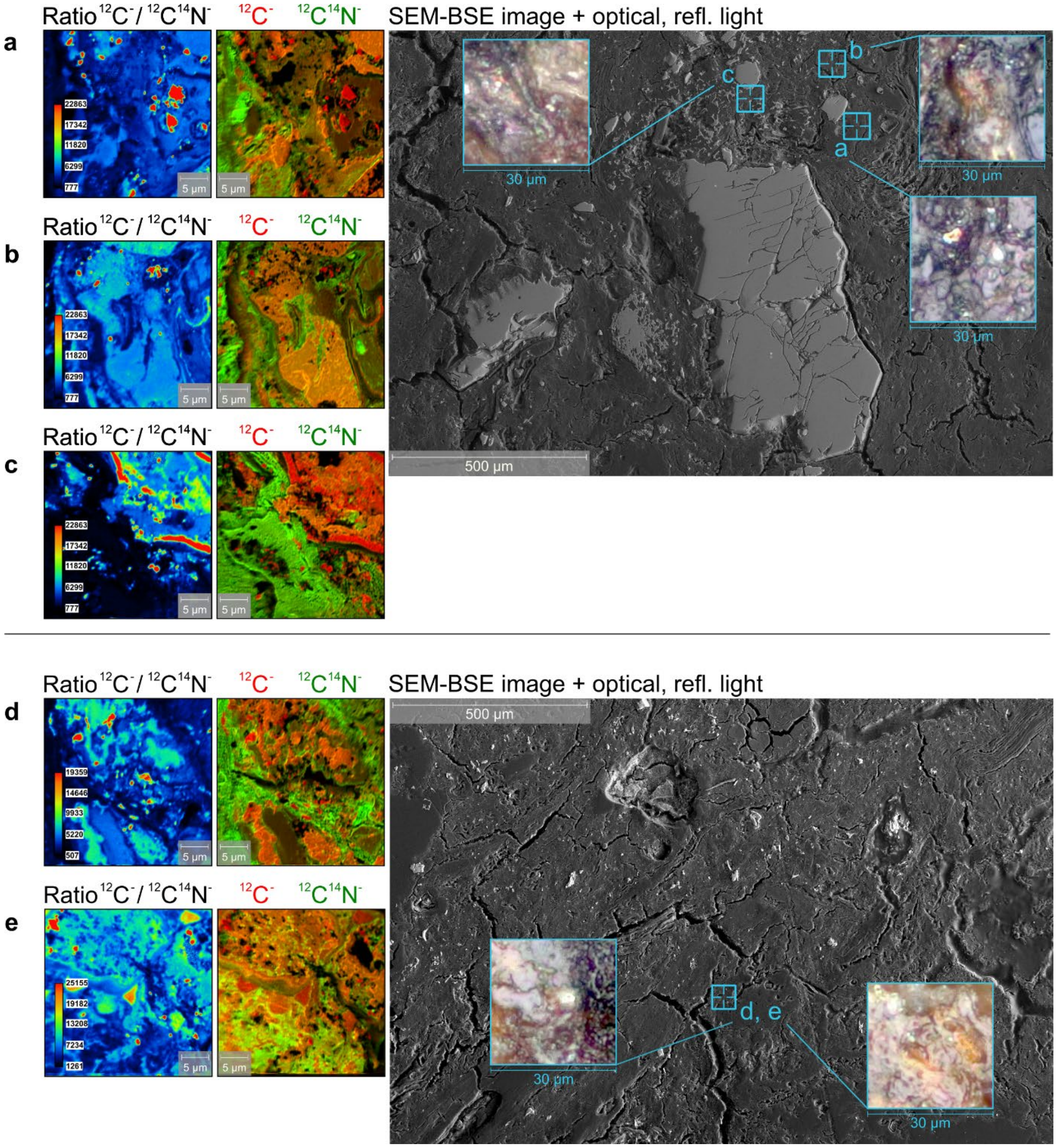

Documentation of NanoSIMS measurements of intra-aggregate components shown in Figs. 5 (a-c) and S7 (d-e). Reflected light microphotographs inserted in SEM micrographs document the selected localities for NanoSIMS operations. In addition, composite images and mass ratios calculated from $^{12}C^-$ and $^{12}C^{14}N^-$ secondary ion data are shown. Composite images and mass ratios were applied to identify areas with abundant resin used for the preparation process of the samples (Figs. S9 and S10). The indicated panel designations refer to them in Figs. 5 and S7.

Fig. S9:

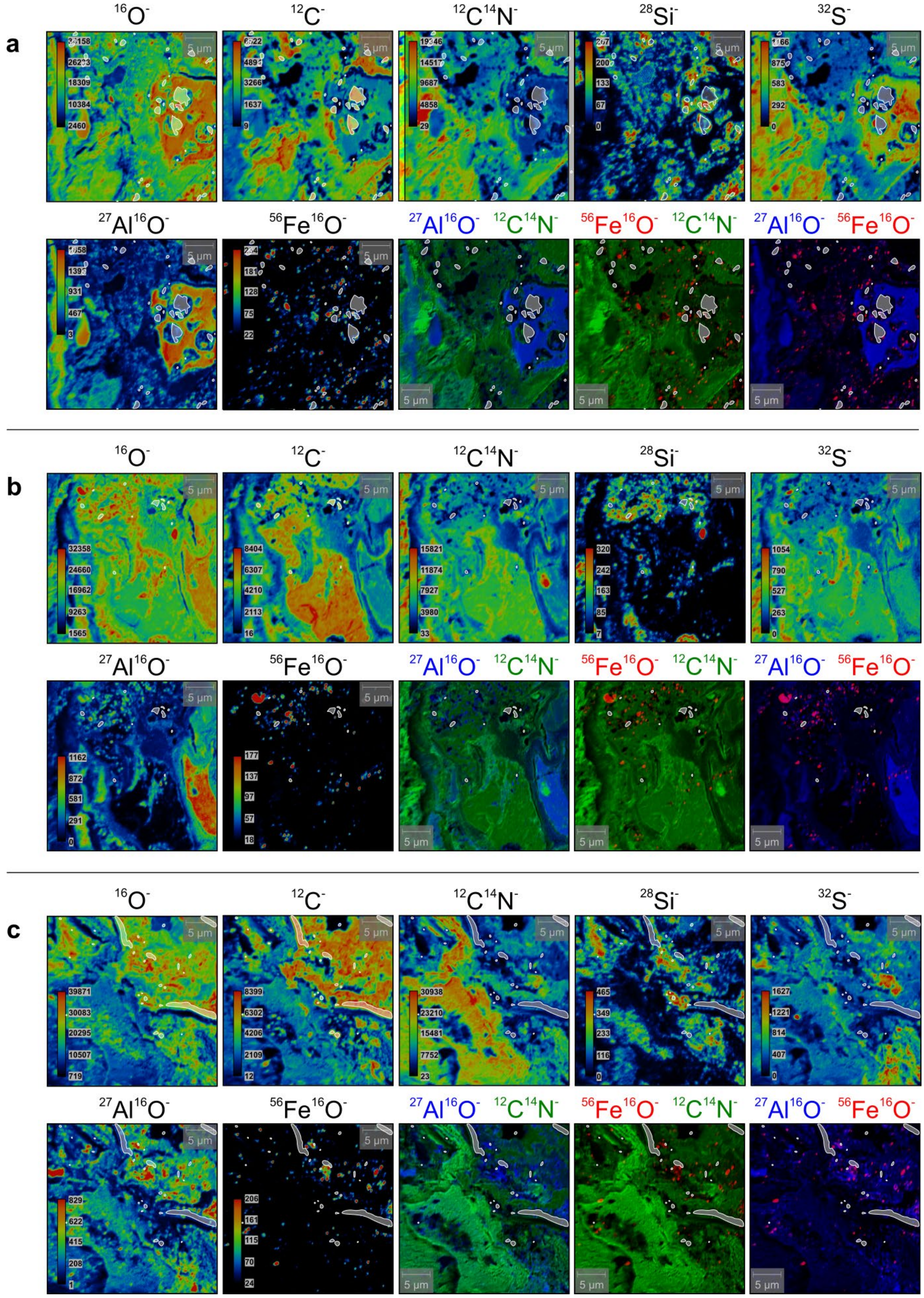

Visualization of the areas containing resin (NanoSIMS secondary ion mappings in Fig. 5) Identified resin is highlighted by white fields on the distribution maps of $^{16}O^-$, $^{12}C^-$, $^{12}C^{14}N^-$, $^{28}Si^-$, $^{32}S^-$, $^{27}Al^{16}O^-$, and $^{56}Fe^{16}O^-$ secondary ions and $^{12}C^{14}N^-$, $^{27}Al^{16}O^-$ and $^{56}Fe^{16}O^-$ composite images. The indicated panel designations refer to them in Fig. 5.

Fig. S10:

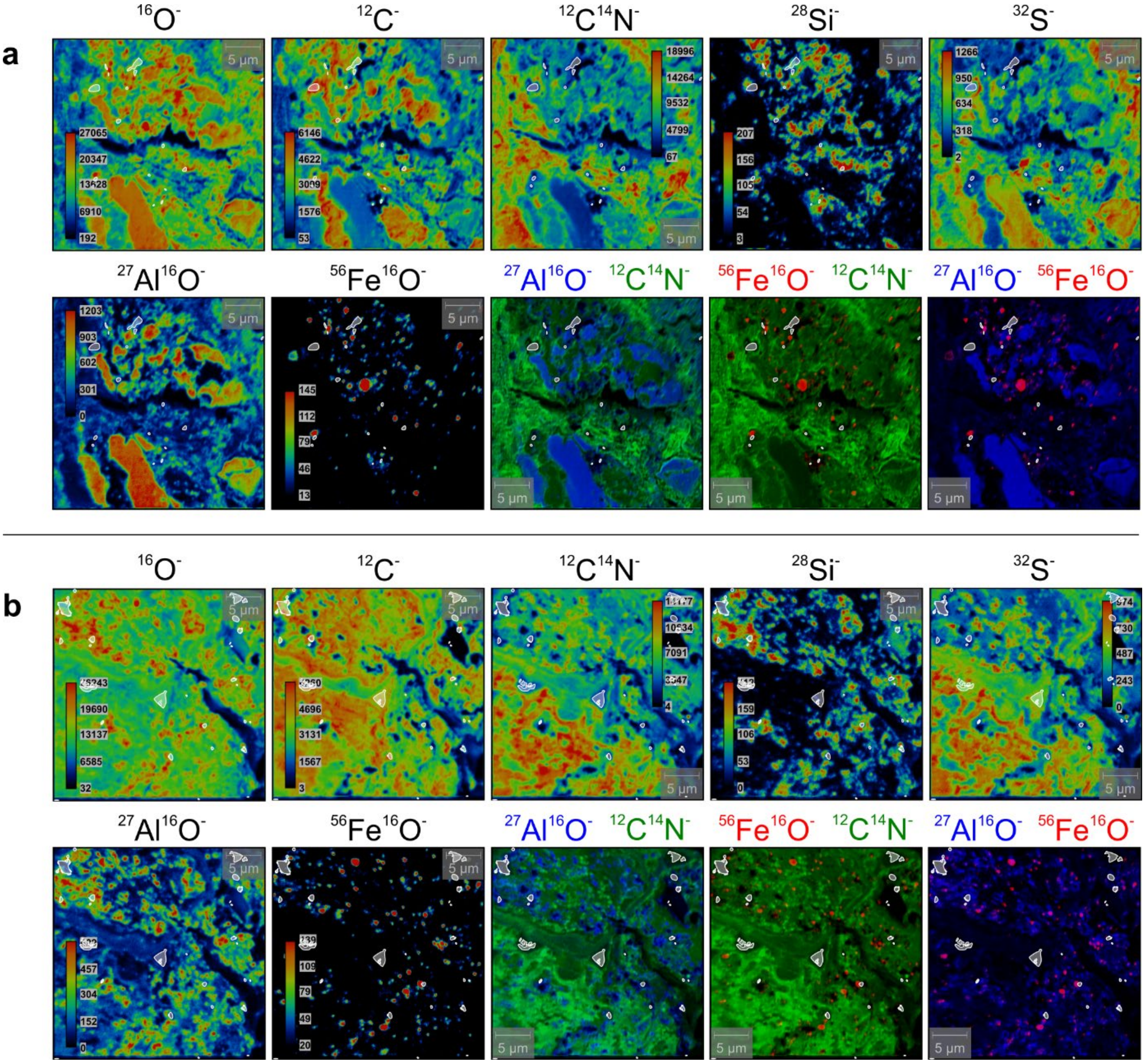

Visualization of the areas containing resin (NanoSIMS secondary ion mappings in Fig. S7) Identified resin is highlighted by white fields on the distribution maps of $^{16}O^-$, $^{12}C^-$, $^{12}C^{14}N^-$, $^{28}Si^-$, $^{32}S^-$, $^{27}Al^{16}O^-$, and $^{56}Fe^{16}O^-$ secondary ions and $^{12}C^{14}N^-$, $^{27}Al^{16}O^-$ and $^{56}Fe^{16}O^-$ composite images. The indicated panel designations refer to them in Fig. S7.

References – Supplementary information

1. Hanesch, M. Raman spectroscopy of iron oxides and (oxy)hydroxides at low laser power and possible applications in environmental magnetic studies. *Geophys. J. Int.* **177**, 941-948 (2009).
2. de Faria, D. L. A., Venacio Silva, S., de Oliveira, M.T. Raman microspectroscopy of some iron oxides and oxyhydroxides. *J. Raman Spectrosc.* **28**, 873-878 (1997).
3. Oh, S. J., Cook, D. C., Townsend, H. E. Characterization of iron oxides commonly formed as corrosion products on steel. *Hyperfine Interact.* **112**, 59-65 (1998).
4. Bridges, J. C. et al. Iron oxides in comet 81P/Wild 2. *Meteorit. Planet. Sci.* **45**, 55-72 (2010).
5. Das, S., Hendry, M. J. Application of Raman spectroscopy to identify iron minerals commonly found in mine wastes. *Chem. Geol.* **290**, 101-108 (2011).
6. Thibeau, R. J., Brown, C. W., Heidersbach, R. H. Raman spectra of possible corrosion products of iron. *Appl. Spectrosc.* **32**, 532-535 (1978).
7. Legodi, M. A., de Waal, D. Raman spectroscopic study of ancient South African domestic clay pottery. *Spetrochim. Acta A. Mol. Biomol. Spectrosc.* **66** (1), 135-142 (2007).
8. Froment, F., Tournié, A., Colomban, P. Raman identification of natural red to yellow pigments: ochre and iron containing ores. *J. Raman Spectrosc.* **39**′, 560-568 (2008).
9. Chourpa, I. et al. Molecular composition of iron oxide nanoparticles, precursors for magnetic drug targeting, as characterized by confocal Raman microspectroscopy. *Analyst* **130**, 1395-1403 (2005).
10. Jacintho, G. V. M., Corio, P., Rubim, J. C. Surface-enhanced Raman spectra of magnetic nanoparticles adsorbed on a silver electrode. *J. Electroanal. Chem.* **603**, 27-34 (2007).
11. Mazzetti, L., Thistlethwaite, P. J. Raman spectra and thermal transformations of ferrihydrite and schwertmannite. *J. Raman Spectrosc.* **33** (2), 104-111 (2002).
12. Müller, K., Ciminelli, V. S. T., Dantas, M. D. S., Willscher, S. A comparative study of As(III) and As(V) in aqueous solutions and adsorbed on iron oxy-hydroxides by Raman spectroscopy. *Water Res.* **44**, 5660-5672 (2010)
13. Weidemann, S. et al. A 17 year record of meteorological observations across the Gran Campo Nevado Ice Cap in Southernmost Patagonia. *Frontiers Earth Sci.* **6** (53), 10.3389/feart.2018.00053 (2018).
14. Peiffer, S. et al. A biogeochemical-hydrological framework for the role of redox-active compounds in aquatic systems. *Nat. Geosci.* **14**, 264-272 (2021).
